# Transcranial Direct Current Stimulation above the Medial Prefrontal Cortex Facilitates Decision-Making following Periods of Low Outcome Controllability

**DOI:** 10.1523/ENEURO.0041-21.2021

**Published:** 2021-09-07

**Authors:** Gábor Csifcsák, Jorunn Bjørkøy, Sarjo Kuyateh, Haakon Reithe, Matthias Mittner

**Affiliations:** Department of Psychology, UiT The Arctic University of Norway, Tromsø 9037, Norway

**Keywords:** decision-making, learned helplessness, medial prefrontal cortex, Pavlovian bias, reinforcement learning, tDCS

## Abstract

Recent studies suggest that choice behavior in reinforcement learning tasks is shaped by the level of outcome controllability. In particular, Pavlovian bias (PB) seems to be enhanced under low levels of control, manifesting in approach tendencies toward rewards and response inhibition when facing potential losses. The medial prefrontal cortex (mPFC) has been implicated both in evaluating outcome controllability and in the recruitment of cognitive control (CC) to suppress maladaptive PB during reinforcement learning. The current study tested whether high-definition transcranial direct current stimulation (HD-tDCS) above the mPFC of healthy humans can influence PB, and counteract the previously documented, deleterious behavioral effects of low outcome controllability on decision-making. In a preregistered, between-group, double-blind study (*N* = 103 adults, both sexes), we tested the interaction between controllability and HD-tDCS on parameters of choice behavior in a Go/NoGo task. Relative to sham stimulation, HD-tDCS resulted in more robust performance improvement following reduced control, an effect that was more pronounced in appetitive trials. In addition, we found evidence for weaker PB when HD-tDCS was administered during low controllability over outcomes. Computational modeling revealed that parameter estimates of learning rate and choice randomness were modulated by controllability, HD-tDCS and their interaction. Overall, these results highlight the potential of our HD-tDCS protocol for interfering with choice arbitration under low levels of control, resulting in more adaptive behavior.

## Visual Abstract

**Figure:**
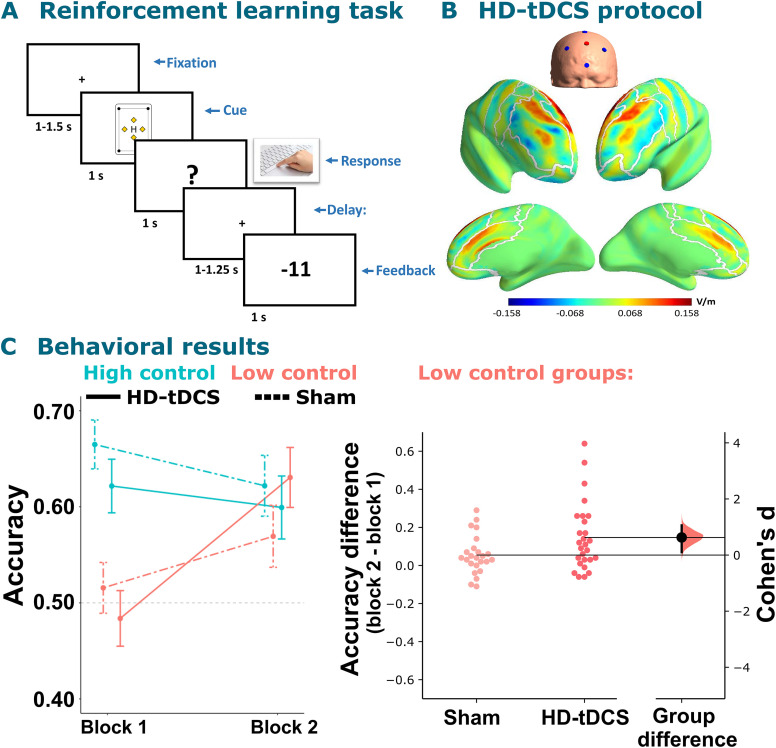

## Significance Statement

Our decisions are shaped by how much control we have over the situation. Under extreme circumstances, low controllability of choice outcomes can lead to learned helplessness (LH) and impaired coping. Since the medial prefrontal cortex (mPFC) was implicated in LH, we tested whether high-definition transcranial direct current stimulation (HD-tDCS) of this region counteracts the deleterious effects of low controllability of rewards and losses in healthy humans. We found stronger improvement in response accuracy when low controllability was combined with HD-tDCS. Moreover, several latent parameters of choice behavior were influenced by HD-tDCS and/or controllability. These results highlight the potential of our HD-tDCS protocol for interfering with choice arbitration in environments with reduced controllability, resulting in more adaptive behavior.

## Introduction

Value-based decision-making is essential for guiding actions toward influencing external events in our favor. Recently, it has been suggested that deliberation strategies can be adjusted to the perceived level of controllability of the environment (Dorfman and Gershman, 2019; Ly et al., 2019). When uncertainty around action outcomes is increased, a commonly used heuristic is to rely more heavily on a Pavlovian bias (PB), manifesting in tendencies for approaching reward-predictive cues, and motor inhibition when facing potential punishment (Rangel et al., 2008; Dayan and Berridge, 2014). A possible explanation for this phenomenon is that the instrumental system relies on more effortful calculation of stimulus-action/action-outcome associations that does not pay off when outcomes are independent of actions. Conversely, Pavlovian stimulus-outcome learning may provide more precise predictions about upcoming events in the absence of response-feedback contingency, which in turn can optimize behavior more cost effectively (Dayan et al., 2006; Rangel et al., 2008; Dorfman and Gershman, 2019).

In extreme cases, the absence of control over aversive events can induce learned helplessness (LH), characterized by anxiety, motor passivity, and impaired decision-making (Pryce et al., 2011; Maier and Seligman, 2016). Once established, LH can also hinder problem solving in new situations with regained control, leading to persistent maladaptive coping. Intriguingly, inaction that is elicited by negative outcomes is a Pavlovian-type response (Rangel et al., 2008; Dayan and Berridge, 2014), raising the possibility that LH is an excessive manifestation of PB in decision-making (Maier and Seligman, 2016). However, the behavioral effects of LH-induction might as well be because of passivity elicited by motor inhibition. To our knowledge, whether low outcome controllability influences Pavlovian response tendencies or whether it facilitates inaction in general, has not been investigated directly in animals.

On the neural level, perceived controllability has been associated with the medial prefrontal cortex (mPFC; Diener et al., 2010; Kerr et al., 2012; Maier and Seligman, 2016; Ly et al., 2019), which regulates activity in subcortical structures as a function of outcome controllability (Amat et al., 2005; Kerr et al., 2012; Maier and Seligman, 2016). In the case of LH, low perceived controllability of negative events can lead to weaker top-down suppression from mPFC toward the dorsal raphe nucleus and the amygdala, both of which have been implicated in defensive behavior and pathologic responses to threat (Amat et al., 2005; Maier and Watkins, 2005; Kerr et al., 2012; Maier and Seligman, 2016; LeDoux and Daw, 2018).

The mPFC also seems to be crucial for mediating the balance between Pavlovian and instrumental responses. This is apparent under Pavlovian conflict, when Pavlovian and instrumental systems promote opposing action policies. For instance, avoiding an appetitive stimulus or approaching large losses can be difficult, since goal-directed aims are in conflict with Pavlovian response tendencies (Guitart-Masip et al., 2012; Hershberger, 1986; Huys et al., 2012). In these situations, cognitive control (CC) linked to the dorsal anterior cingulate cortex (dACC) was proposed as a mechanism for suppressing maladaptive PB, and consequently, to optimize behavior (Cavanagh et al., 2013; Cavanagh and Frank, 2014; Swart et al., 2018). A recent study provided a more direct link between PB, controllability and dACC activity, by showing that intermittent absence of control over rewards and losses during reinforcement learning enhanced PB, and interfered with the neurophysiological correlate of CC, arising from dACC (Csifcsák et al., 2020). The authors concluded that manipulation of controllability levels can influence the magnitude of CC over PB in action selection.

The aims of the current study were twofold. First, using a new controllability manipulation, we wished to extend knowledge on the effect of low outcome controllability on PB and response accuracy during reinforcement learning (Dorfman and Gershman, 2019; Csifcsák et al., 2020). We hypothesized that, relative to a control condition, low controllability of outcomes would result in stronger PB and worse performance on Pavlovian-conflict trials. We anticipated that these effects, if sufficiently strong, would outlast the period of controllability manipulation, and manifest in a transfer to the subsequent block, where control over rewards and losses is restored.

**Figure 1.**
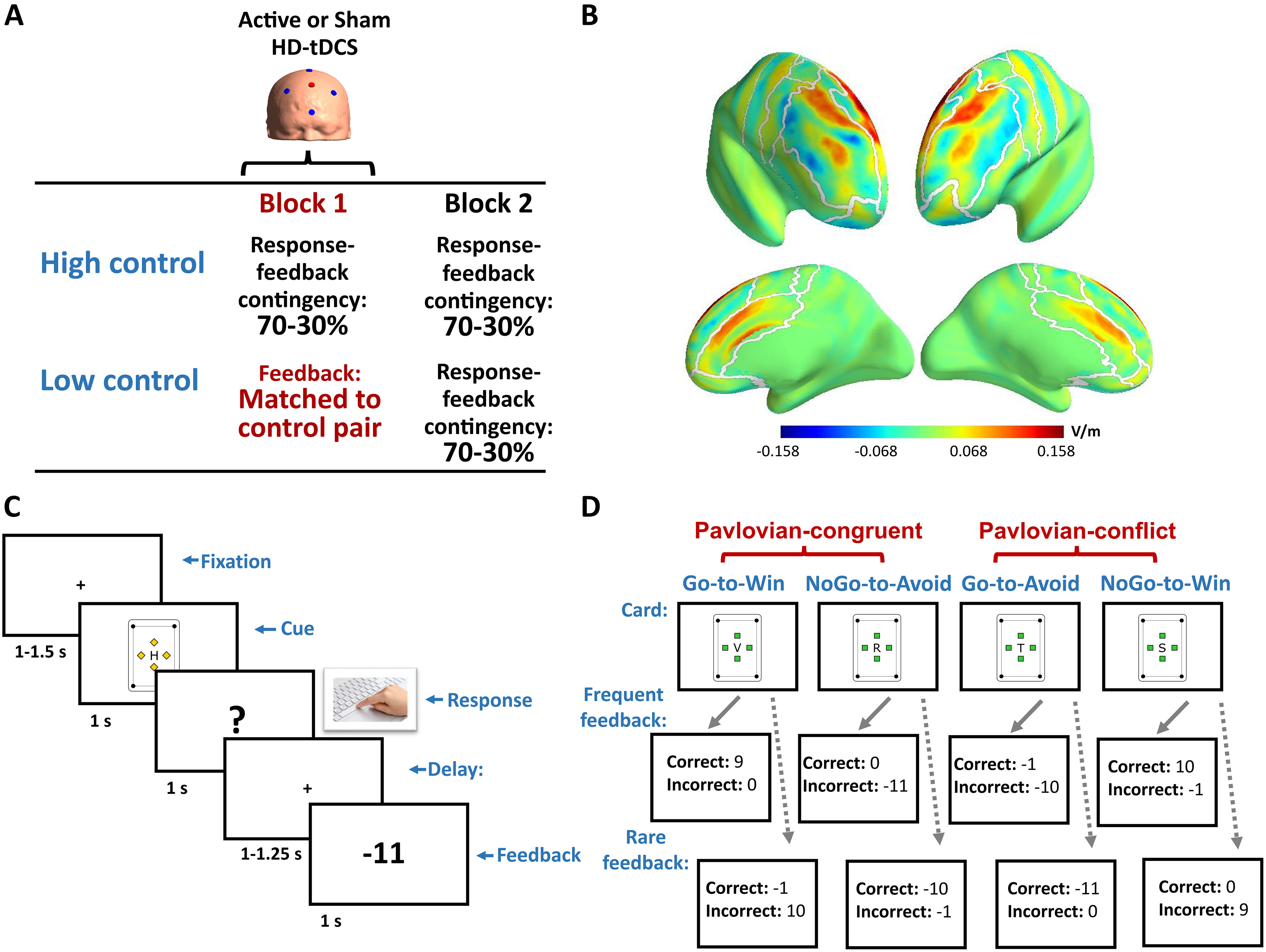
Overview of our study design (***A***), the spatial distribution and magnitude of the normal components of HD-tDCS-induced electric fields, representing currents either entering or leaving the cerebral cortex (depicted with positive and negative values, respectively), averaged across 18 head models of healthy adults (***B***), trial structure (***C***), and card types with feedback values (***D***).

Our second aim was to test whether high-definition transcranial direct current stimulation (HD-tDCS) above the mPFC reverses the behavioral consequences of low controllability (Fig. 1*A*). Whereas conventional tDCS montages use large electrodes that are placed further apart from each other, HD-tDCS consists of several, closely arranged small electrodes that provide more focal stimulation (Datta et al., 2009). We selected a “4 × 1” HD-tDCS protocol that provides, according to simulation studies (Csifcsák et al., 2018), relatively circumscribed and predominantly excitatory stimulation in the mPFC, potentially even reaching the dACC (Fig. 1*B*).

To investigate the interaction between controllability and HD-tDCS, we adopted a double-blind, between-group design following a preregistered protocol (https://osf.io/h45ju). Concerning our hypothesis about the effect of HD-tDCS, we predicted that, relative to the sham protocol, stimulation of the mPFC/dACC would enhance CC, resulting in weaker PB and improved response accuracy both during and following HD-tDCS. In this respect, we postulated that HD-tDCS would counteract the deleterious effects of low outcome controllability on task performance.

## Materials and Methods

### Participants

Human subjects were recruited via public advertisements in Tromsø, Norway. Based on our a priori power analysis [repeated-measures (rm)ANOVA, Cohen’s *f *=* *0.2 for the within-between factor interaction for block × group, 1-*β* = 0.9, *α* = 0.05, minimum required sample size = 96], 104 healthy adults from both sexes signed the informed consent and were randomized to one of four groups differing in the level of outcome controllability (high vs low control) and HD-tDCS (stimulated vs sham). Data from one participant was discarded because of technical errors, yielding 103 participants (HighControl-Stimulated: *N* = 26, HighControl-Sham: *N* = 26, LowControl-Stimulated: *N* = 27, LowControl-Sham: *N* = 24; 64 females). We had to deviate from our prespecified exclusion criteria (i.e., excluding participants not producing at least one Go and one NoGo response to all four card types in both blocks) because an impractically high number of participants (*N* = 50, evenly distributed across groups) had to be excluded from further analysis using this criterion. This change was made before any formal data analysis took place and should therefore not compromise our preregistered analyses. A possible explanation for the high number of subjects not producing both response options to all cards in each block may be that 14 participants showed excessive PB (predominantly in block 1), whereas another 19 individuals could very successfully suppress their PB (mainly in block 2), leading to ceiling/floor effects in terms of response accuracy for some cards. However, we note that all participants in the final sample produced at least one Go and one NoGo response in both blocks. The groups did not differ in age (M = 23.3 years, SD = 2.7, *F*_(3,99)_ = 0.61, *p *=* *0.606) or sex (*H*_(3)_ = 2.08, *p *=* *0.555). Participants received gift cards worth ∼22.5 USD. The study protocol complied with the Declaration of Helsinki and was approved by the Institutional Ethics Committee. All data and study materials are available at https://osf.io/d6eqk/.

### Study design

First, a local anesthetic cream containing lidocaine/prilocaine (“EMLA”) was distributed at electrode locations to ensure proper blinding. Next, we collected data on mood in the past month (PANAS-Past) and at the moment (PANAS-Present-Before; Watson et al., 1988), personality attributes of motivated behavior (BIS/BAS; Carver and White, 1994) and predisposition to develop hopelessness (BHS; Beck et al., 1974). Participants read task instructions (framed as a card game), performed a short practice session, and completed a quiz to ensure they understood all important aspects of the game. Quiz items with wrong answers were re-visited and discussed. Subsequently, we placed the electrode cap with electrodes and a small amount of conductive gel on the head of participants, and made sure that impedances were below 10 kΩ.

The task consisted of two task blocks with a response-feedback contingency of 70/30%, except for block 1 in participants in the LowControl groups. Real or sham HD-tDCS was also delivered during block 1, using a prespecified double-blinded protocol. After each block, participants rated their perceived levels of success and control using two visual analog scales (data missing for one participant). At the end of the session, participants had to guess whether they received real or sham stimulation, which was followed by assessing momentary mood scores (PANAS-Present-After) and a working memory task (OSPAN; Turner and Engle, 1989).

### Task and controllability manipulation

We used the modified version of the orthogonalized Go/NoGo task that was designed to investigate the neural correlates of PB during instrumental learning (Cavanagh et al., 2013; Guitart-Masip et al., 2012). Participants had to collect points by learning whether to respond (Go: “pick up”) or not (NoGo: “leave on the table”) to each card. They were informed that there would be “winning” and “losing” cards, and that Win cards would either provide a reward (10 points) or zero outcome, whereas Avoid cards could result in a loss (−10 points) or the absence thereof. Participants were also aware that favorable versus unfavorable outcomes were determined by correct versus incorrect responses, albeit in a probabilistic manner, with occasional “misleading outcomes.” Outcomes were penalized by a “Go-cost” (−1 point) if they were preceded by a Go response. Therefore, following an active response, win, no win/no loss and loss outcomes were modified to 9, −1, and −11 points, respectively. The Go-cost was framed as the cost/effort of exploring by action, mimicking real-life situations (Teodorescu and Erev, 2014). The task consisted of two experimental blocks consisting of 160 trials each (four cards × 40 repetitions).

For running the task, we used a desktop computer with Windows XP Professional operating system, Intel (R) Core(TM)2 Duo CPU, 2.33 GHz, 1.96 GB RAM, and a 19-inch Sony Trinitron CRT monitor with 1024 × 768 resolution and 100 Hz refresh rate. Stimuli were presented and responses were collected using PsychoPy 1.83.04 (Peirce, 2007). Trials started with a central fixation sign, followed by a custom-made card, the response screen, a short delay and the outcome (Fig. 1*C*). Participants were asked to respond only when the question mark appeared on the screen, which always occurred 1 s after cue onset. Thus, the current task design did not enable assessing reaction times. In each block, four new cards were shown (Go-to-Win, NoGo-to-Avoid, Go-to-Avoid, NoGo-to-Win), depending on their valence (reward vs loss) and action requirement (Go vs NoGo; Fig. 1*D*). Given that the Pavlovian system promotes approach toward rewards and inhibits response tendencies for losses, Go-to-Win and NoGo-to-Avoid cards were Pavlovian-congruent, whereas Go-to-Avoid and NoGo-to-Win cards were associated with Pavlovian conflict.

We aimed to induce helplessness by manipulating action-outcome contingency in the LowControl groups. Unbeknownst to the participants, each LowControl individual was paired with a HighControl participant. HighControl versus LowControl pairs were created by counterbalancing HD-tDCS conditions. For each HighControl participant, we recorded the outcomes from block 1 for the four card types separately, but removing the effect of the Go-cost when appropriate. These outcomes were shown in a random order, but in a card-specific manner to the corresponding paired subject in the LowControl group. That is, outcomes for a HighControl participant’s Go-to-Win card were presented to the matched participant from the LowControl group for the card that was also labeled as Go-to-Win. Our manipulation ensured that controllability over rewards and losses was absent in this block (except for the Go-cost), while matching reward/loss frequency between groups. Importantly, manipulated outcomes were penalized by a Go-cost based on LowControl participants’ own responses, leading to a possible discrepancy in reward/loss magnitude between HighControl and LowControl groups. For instance, a Go-associated reward (nine points) for a HighControl participant could be modified to 10 points if the same outcome was presented following a LowControl participant’s NoGo response (or vice versa). By keeping the Go-cost during controllability manipulation we aimed for promoting behavioral passivity and limiting active exploration, which are key features of helplessness (Maier and Seligman, 1976; Teodorescu and Erev, 2014). However, it is important to note that the Go-cost provided some level of outcome controllability in block 1 to the LowControl groups, by reducing each outcome on active responding.

### HD-tDCS

Brain stimulation was delivered with a Starstim device, using neoprene headcaps, conductive gel (SignaGel) and Ag/AgCl electrodes with a diameter of 12 mm (Neuroelectrics). Electrodes were placed at scalp positions Fpz, Fz, Cz, F3, and F4, with Fz serving as anode (2 mA) and the surrounding four electrodes as returns (0.5 mA each). The choice of the electrode montage was based on our simulations of HD-tDCS-induced electric fields, using 18 realistic head models of healthy adults, downloaded from a freely available database (https://osf.io/exbd5/; Boayue et al., 2018). Simulations were performed with the freely available SimNIBS software (Thielscher et al., 2015). We chose to evaluate the spatial distribution and magnitude of the normal component of electric fields, since it represents currents either entering or leaving the pial surface of the cortex, associated with predominantly excitatory or inhibitory effects (Rahman et al., 2013). Our montage yielded facilitatory currents in the superior-lateral and medial surfaces of the PFC in both hemispheres, possibly even reaching the dACC (Fig. 1*B*).

Real stimulation consisted of 30-s ramp-up, 15 min stimulation and 30-s ramp-down, whereas the sham session only contained two 30-s ramp-up/ramp-down periods at the beginning and end of a 16 min period, with no stimulation in-between. Neither the experimenters, nor the participants were aware of group assignment (double-blind protocols), and the percentage of participants guessing that they received real stimulation was comparable across groups, indicating proper blinding (HighControl-Stimulated: 34.6%, HighControl-Sham: 50.0%, LowControl-Stimulated: 55.5%, LowControl-Sham: 60.8%; *H*_(3)_ = 3.84, *p *=* *0.279).

### Preregistered analysis

Our primary focus was the change in PB across experimental blocks and groups. Therefore, we calculated the Pavlovian performance index (PPI) as the mean of two measures, reward-based invigoration (the number of Go responses on win trials/total number of Go) and punishment-based suppression (the number of NoGo responses on avoid trials/total number of NoGo). These indices represent the likelihood to initiate actions toward rewards and inhibit responses when facing potential loss, respectively (Cavanagh et al., 2013). We also calculated response accuracy as the ratio of correct responses for each block and card type.

PPI and accuracy were entered into rmANOVA with group as between-subject and block as within-subject factors, and additional within-subject factors of card congruency and valence for accuracy. Main effects and interactions were interpreted as significant at *p* < 0.05. Estimates of effect size (η_p_^2^) are also reported. Furthermore, Cumming estimation plots were used to illustrate effect sizes for pairwise comparison of conditions, whenever appropriate (Ho et al., 2019). We note, however, that Cumming estimation plots were not included in our preregistered analysis pipeline, and we used them to verify and/or extend results from rmANOVA. We chose estimation statistics because they provide robust estimates about the underlying effect sizes with resampling-based confidence intervals (CIs). Thus, this approach avoids pitfalls of dichotomous significance testing by focusing on the magnitude of effects, while also accounting for the precision of the estimation method using bias-corrected and accelerated bootstrapping (Ho et al., 2019).

### Computational modeling

To gain a more nuanced view on the effects of our interventions on latent processes of reinforcement learning and decision-making, we also implemented a computational model to our behavioral data. Previous studies on Pavlovian-instrumental interactions have successfully applied such models to extract various parameters of choice behavior, and showed that this approach unravels hidden associations that cannot be captured by more conventional data analysis (Guitart-Masip et al., 2012; Cavanagh et al., 2013; Swart et al., 2018; Csifcsák et al., 2020). For computational modeling, we used a Precision 7920 Rack computer, Debian GNU/Linux 9.9 operating system, 2× Intel Gold 6152, 2.1 GHz, 22 cores, and 512 GB RAM. Our primary interest was to look for potential group differences in the temporal evolution of the PB parameter *π*, but we also extracted parameters representing randomness of choice (temperature; *β*), learning rate (*α*) and the general tendency to initiate actions (Go-bias; *b_go_*). This approach was very similar to those used in previous studies (Cavanagh et al., 2013; Swart et al., 2018; Csifcsák et al., 2020), with the exception that our model did not incorporate single-trial EEG data. All four parameters capture choice behavior from a different perspective. Changes in the Pavlovian parameter (*π*) were expected to corroborate findings on PPI, with higher values in LowControl participants receiving sham stimulation, but a reduction during/following HD-tDCS. With respect to randomness of choice (*β*), learning rate (*α*), and Go-bias (*b_go_*), our analysis was more exploratory, aiming at providing supportive evidence to a study (Csifcsák et al., 2020) reporting increased values for all three parameters during manipulated outcome controllability.

Action choices (Go vs NoGo) for subject i in trial t of block j for stimulus $s_{t}$ were modelled with the Bernoulli-experiment with probabilities P(Go) and P(NoGo)=1−p(Go) as

(1) $$P(\text{Go}|s_{t,j,i})=\frac{\text{exp}\left[W_{t}(\text{Go}|s_{t,j,i})/\beta_{j,i}\right]}{\text{exp}\left[W_{t}(\text{Go}|s_{t,j,i})/\beta_{j,i}\right]+\text{exp}\left[W_{t}(\text{NoGo}|s_{t,j,i})/\beta_{j,i}\right]},$$

where $W_{t}$ is response weight (Go vs NoGo) of the stimulus, and temperature parameter $\beta_{j,i}$ determines how biased the decisions are in favor of the higher-weighted option. For a given stimulus/action value $Q_{t}$,

(2) $$W_{t}(a|s_{t,j,i})=\{\begin{array}{ll} Q_{t}(\text{Go}|s_{t,j,i})+b_{j,i}+\pi_{j,i}V(s_{t,j,i}) & \text{if }a=\text{Go}\\ Q_{t}(\text{NoGo}|s_{t,j,i}) & \text{if }a=\text{NoGo} \end{array},$$

where parameter $b_{j,i}$ codes for a general Go-bias, and $\pi_{j,i}$ is our crucial PB parameter that scales learnt stimulus value $V(s_{t,j,i})$ in a way that it favors action/inaction for win/avoid cards. The value of stimulus $s_{t,j,i}$ is cumulated as

(3) $$V(s_{t,j,i})=V_{t-1}(s_{t,j,i})+\alpha_{j,i}\left[r_{t,i,j}-V_{t-1}(s_{t,j,i}))\right],$$

where $\alpha_{j,i}$ is the learning rate and $r_{t,j,i}$ is the reward (feedback). The final bit of the model is a standard Q-learning mechanism where stimulus/action pairs receive a value $Q_{t}(a|s_{t,j,i})$ that are updated to the standard rule

(4) $$Q_{t}(a|s_{t,j,i})=Q_{t-1}(a|s_{t,j,i})+\alpha_{j,i}\left[r_{t,j,i}-Q_{t-1}(a|s_{t,j,i}))\right].$$

We model the data from all subject and sessions in the framework of hierarchical Bayesian modeling. We refer the reader to Gelman et al. (2003) for in-depth coverage of the advantages of this approach. All models where implemented using Hamiltonian Monte Carlo algorithms (Hoffman and Gelman, 2014) implemented in Stan (Carpenter et al., 2017). We used six parallel chains with warm-up period of 1000 samples each such that 6000 samples were drawn from the converged chains. Traceplots for all variables were manually screened for convergence. In addition, we calculated the Gelman–Rubin diagnostic (Gelman and Rubin, 1992) to ensure that all $\hat{R}\leq 1.05$.

The dependency of each model parameter on block, controllability manipulation, HD-tDCS, and their interactions were included at the group-level in the hierarchical model directly. Posterior densities for the estimated coefficients were calculated and regarded as relevant if their 95% highest density interval (HDI) excluded zero. When reporting regression coefficients, we report posterior mean *b*, 95% HDI and the evidence ratio (ER) in favor of a positive (ER_+_) or a negative effect (ER_–_). ER can be interpreted as an odds ratio, calculated as the ratio of two probabilities: the probability of the effect being positive, *P(b > 0)*, divided by the inverse probability of the effect being zero or negative, *1-P(b > 0)*, for ER_+_ or its inverse for ER_–_. For example, the statement *b *=* *0.09 [0.01, 0.18], ER_+_ = 27.0 indicates that it is 27 times as likely that the effect is positive than that it is zero or negative. In this analysis, *P* values represent posterior probabilities that values are either below or above zero, and are not to be confused with frequentist *p* values.

### Code accessibility

The code/software described in the paper is freely available online at https://osf.io/d6eqk/.

## Results

### Preregistered analysis

**Figure 2.**
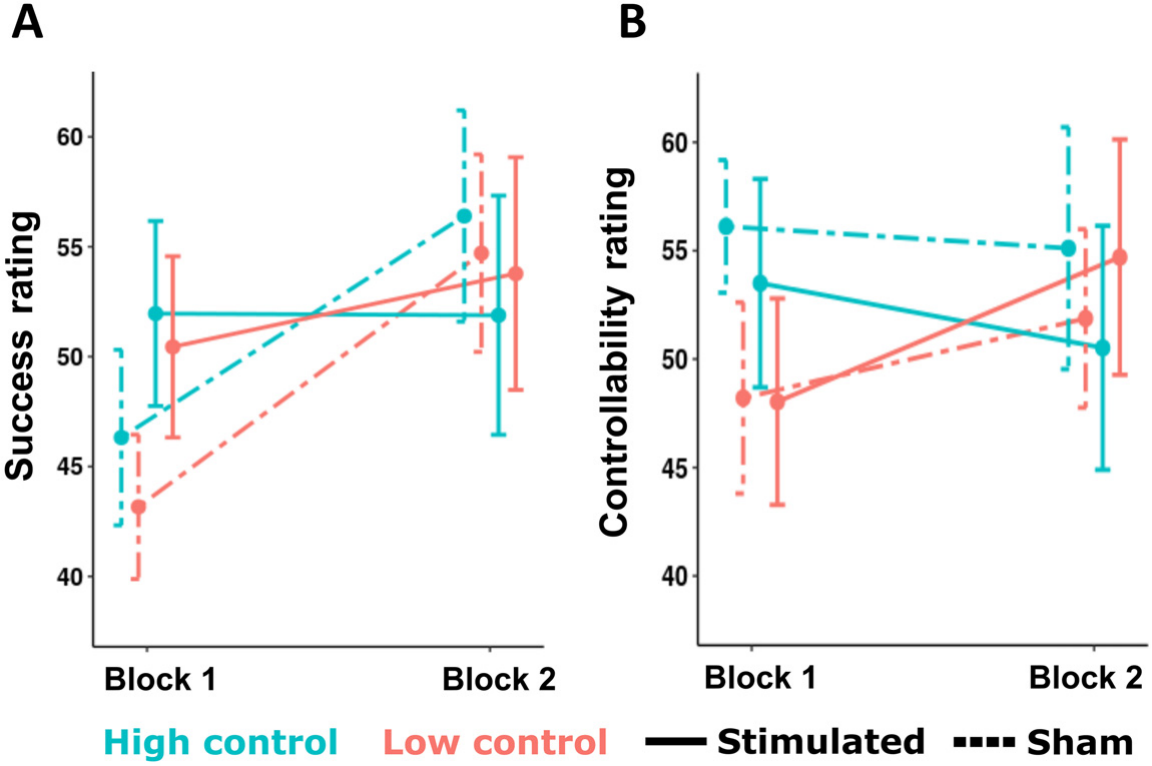
Ratings (means and SEs) of perceived success (***A***) and outcome controllability (***B***) following each block.

Statistical analysis of questionnaire data and working memory performance collected at baseline are presented in Table 1. Experimental groups did not differ in their past mood (PANAS-Past), hopelessness (BHS), personality traits (BIS/BAS), or working memory capacity (OSPAN). Statistical results concerning repeated measurements of momentary mood ratings (PANAS-Present) revealed reduced scores by the end of the session, but no group differences were found (Table 2). Subjective ratings of perceived success increased by block 2, but scores were statistically comparable across groups (Table 2; Fig. 2*A*). As for perceived outcome controllability, no main effects or interactions were found (Table 2; Fig. 2*B*).

**Table 1:** Statistical results for the comparison of questionnaire data and cognitive tests between the four experimental groups

Baseline measures	Group effect			
	*F*	df	*p*	η_p_^2^
PANAS-Pa-Pos	0.43	3,99	0.731	0.013
PANAS-Pa-Neg	0.17	3,99	0.916	0.005
BIS/BAS	0.49	3,99	0.691	0.015
BHS	0.46	3,99	0.710	0.014
OSPAN	0.14	3,96	0.933	0.004

BIS/BAS: behavioral inhibition/approach system; BHS: Beck hopelessness scale; OSPAN: operation span task; PANAS-Pa-Neg: negative past mood scores on the positive and negative affect schedule; PANAS-Pa-Pos: positive past mood scores on the positive and negative affect schedule.

**Table 2:** Statistical results for the comparison of questionnaire data and subjective ratings between the four experimental groups and the repeated measurements

	Group effect				Block effect				Block × group interaction			
	*F*	df	*p*	η_p_^2^	*F*	df	*p*	η_p_^2^	*F*	df	*p*	η_p_^2^
PANAS-Pr-Pos	0.37	3,98	0.820	0.009	23.99	1,98	**<0.001**	0.197	1.75	3,98	0.162	0.05
PANAS-Pr-Neg	0.61	3,98	0.611	0.018	10.99	1,98	**0.001**	0.101	1.33	3,98	0.267	0.04
Success rating	0.12	3,98	0.949	0.004	12.27	1,98	**<0.001**	0.111	2.39	3,98	0.073	0.07
Control rating	0.81	3,98	0.488	0.024	0.80	1,98	0.373	0.008	0.81	3,98	0.492	0.024

PANAS-Pr-Neg: negative momentary mood scores on the positive and negative affect schedule; PANAS-Pr-Pos: positive momentary mood scores on the positive and negative affect schedule. Significant (*p* < 0.05) effects are highlighted with bold.

**Figure 3.**
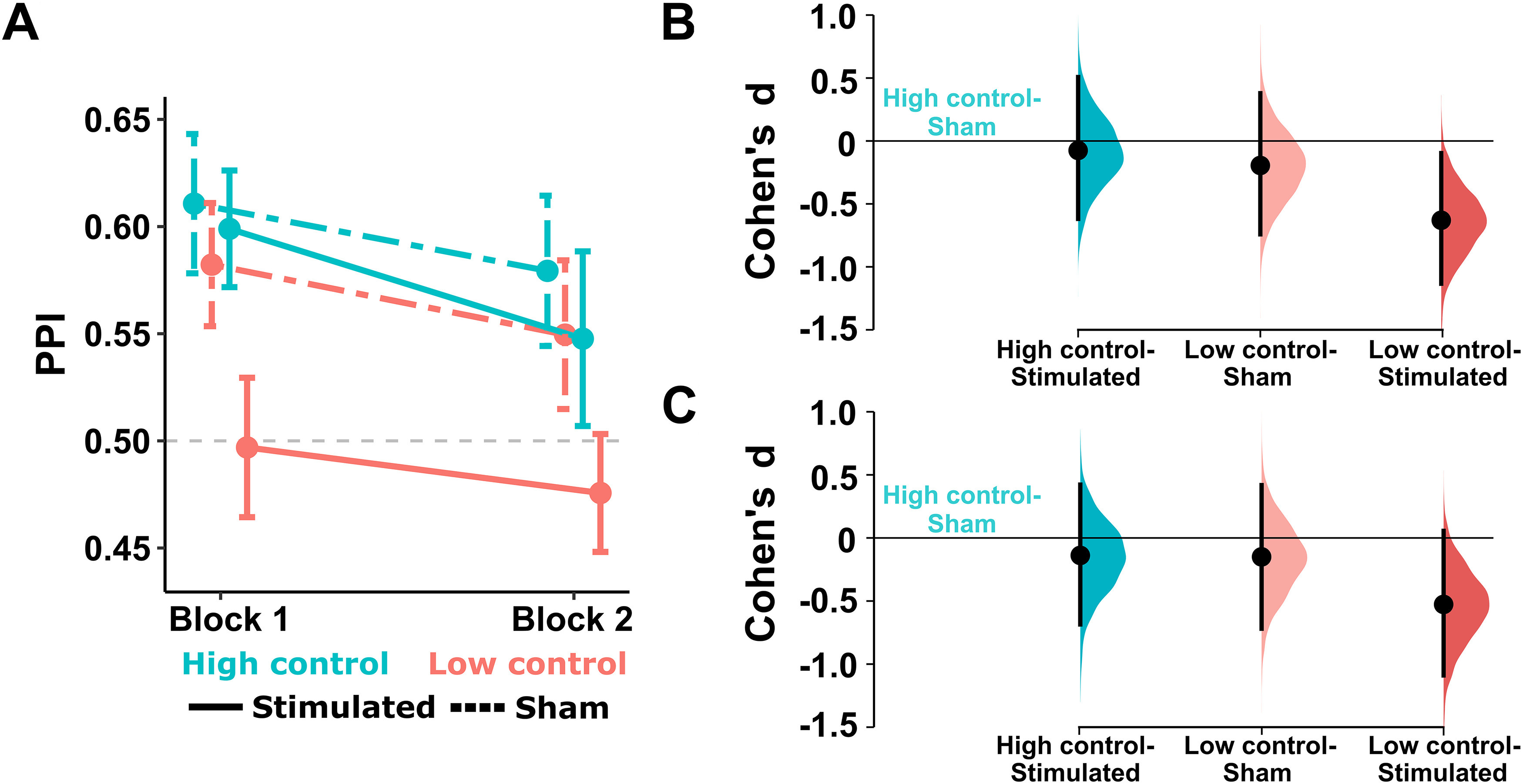
Changes in the magnitude of the PPI (means and SEs) across the two blocks and four experimental groups (***A***), and Cumming estimation plots showing effect size estimates (Cohen’s *d*) for three comparisons against a shared control condition (HighControl-Sham group) for block 1 (***B***) and block 2 (***C***). Mean differences are presented as black dots, along with the corresponding bootstrap sampling distributions (5000 samples) and the bias-corrected and accelerated 95% CIs (black bars). Raw data and Cumming estimation plots related to changes from block 1 to block 2 for each group are presented in Extended Data Figure 3-1.

With respect to our PB measure, PPI, a significant reduction in block 2 confirmed that participants gradually learned to suppress their PB (block: *F*_(1,99)_ = 3.99, *p *= 0.048, η_p_^2^ = 0.039). However, PPI was not influenced by group membership, despite showing lower values in the crucial LowControl-Stimulated group (group: *F*_(3,99)_ = 2.11, *p *=* *0.104, η_p_^2^ = 0.060; block × group: *F*_(3,99)_ = 0.14, *p *=* *0.937, η_p_^2^ = 0.004; Fig. 3*A*). Paired data from all individuals along with the effect size estimates (bootstrapped 95% CIs) for changes in PPI from block 1 to block 2 are shown in Extended Data Figure 3-1, indicating comparable Block-effects across the four groups. Crucially, Cumming estimation plots for the effect size (Cohen’s *d*) obtained for the comparison of PPI values from the three groups receiving experimental interventions (HighControl-Stimulated, LowControl-Sham, LowControl-Stimulated) against a shared control (HighControl-Sham) showed reduced PPI only in the LowControl-Stimulated group in block 1, quantified by a medium mean effect size (mean = −0.63, 95% CI = [−1.13, −0.01]; Fig. 3*B*). A similar trend was observed in block 2, although the 95% CI did not exclude zero (−0.52 [−1.09, 0.05]; Fig. 3*C*). Finally, we assessed whether the two sub-measures of PPI, reward-based invigoration and punishment-based suppression were similarly influenced by our interventions. This analysis yielded largely similar results to PPI (estimates for Cohen’s *d* for the LowControl-Stimulated vs HighControl-Sham comparison: reward-based invigoration in block 1: −0.59 [−1.1, −0.06], block 2: −0.48 [−1.02, 0.08], punishment-based suppression in block 1: −0.62 [−1.12, −0.09], block 2: −0.55 [−1.1, 0.05]). For all other comparisons, mean effect size estimates were substantially weaker (between −0.27 and −0.04), and 95% CIs always included zero. Overall, these findings provide some evidence for the efficacy of HD-tDCS in reducing PB when mPFC stimulation occurs simultaneously with the absence of control over rewards and losses.

10.1523/ENEURO.0041-21.2021.f3-1 Extended Data Figure 3-1 Raw data (upper panel) showing changes in the PPI from block 1 to block 2 for each participant, and Cumming estimation plots (lower panel) representing effect size estimates (Cohen’s *d*) for the change in response accuracy from block 1 to block 2, plotted separately for the four groups. Mean differences are presented as dots, along with the corresponding bootstrap sampling distributions (5000 samples) and the bias-corrected and accelerated 95% CIs. Download Figure 3-1, TIF file.

**Figure 4.**
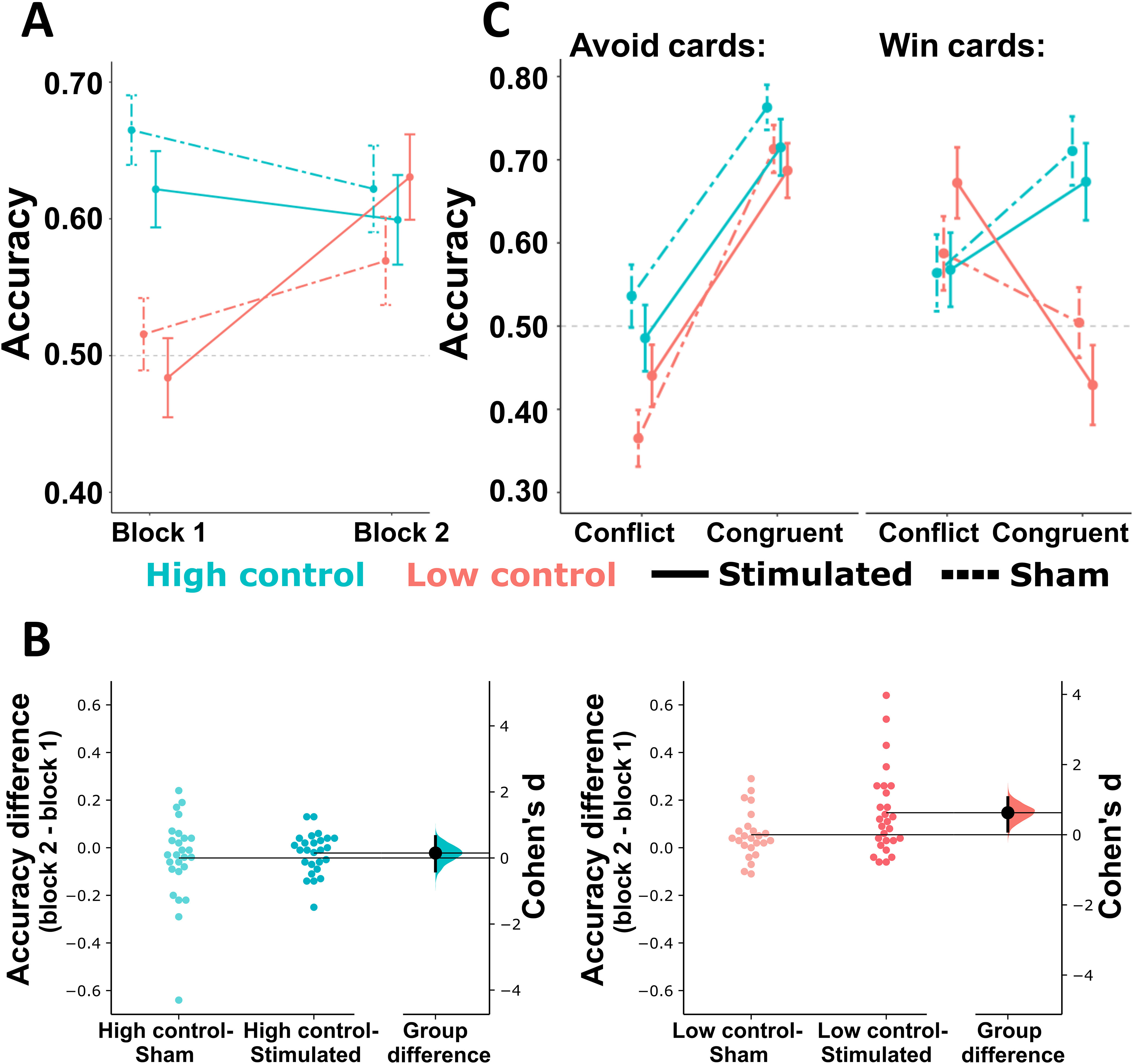
Response accuracy (means and SEs) in each block and experimental group (***A***). Data corresponding to accuracy for each card type, block and group are presented in Extended Data Figure 4-1. Cumming estimation plots representing effect size estimates (Cohen’s *d*) for the pairwise comparison of block-effects between groups receiving real versus sham HD-tDCS, calculated separately for HighControl and LowControl groups are shown in ***B***. Mean differences are presented as black dots, along with the corresponding bootstrap sampling distributions (5000 samples) and the bias-corrected and accelerated 95% CIs (black bars). Raw data and Cumming estimation plots related to changes from block 1 to block 2 for each group are presented in Extended Data Figure 4-2. Changes in response accuracy (means and SEs) corresponding to the four groups and Pavlovian-conflict versus Pavlovian-congruent cards are plotted separately for Avoid and Win cards (***C***). Extended Data Figure 4-3 shows the same interaction between card valence, Pavlovian congruency and group, plotted separately for the two blocks. Extended Data Figure 4-4 shows the percentage of Go responses (PercGo) separately for each card, group and experimental block.

Analysis of response accuracy revealed significant main effects for congruency (*F*_(1,99)_ = 16.60, *p *<* *0.001, η_p_^2^ = 0.144), block (*F*_(1,99)_ = 5.72, *p *=* *0.019, η_p_^2^ = 0.055), and group (*F*_(3,99)_ = 4.52, *p *=* *0.005, η_p_^2^ = 0.120). Importantly, the significant block × group interaction (*F*_(3,99)_ = 9.60, *p *<* *0.001, η_p_^2^ = 0.225) was because of significantly improved responding from block 1 to block 2 in the LowControl-Stimulated group only (*p_Bonferroni_* < 0.001; *p_Bonferroni_* > 0.068 for other groups; Fig. 4*A*), with only this group showing increased mean response accuracies by block 2 for all four card types (Extended Data Fig. 4-1). As a result of low controllability, both LowControl groups produced significantly worse response accuracies in block 1 relative to HighControl groups (*p_Bonferroni_* < 0.023 for all comparisons; Fig. 4*A*).

10.1523/ENEURO.0041-21.2021.f4-1 Extended Data Figure 4-1 Response accuracy (means and SEs) across the two blocks and four experimental groups, plotted separately for the four card types. Download Figure 4-1, TIF file.

10.1523/ENEURO.0041-21.2021.f4-2 Extended Data Figure 4-2 Raw data (upper panel) showing changes in response accuracy from block 1 to block 2 for each participant, and Cumming estimation plots (lower panel) representing effect size estimates (Cohen’s *d*) for the change in response accuracy from block 1 to block 2, plotted separately for the four groups. Mean differences are presented as dots, along with the corresponding bootstrap sampling distributions (5000 samples) and the bias-corrected and accelerated 95% CIs. Download Figure 4-2, TIF file.

10.1523/ENEURO.0041-21.2021.f4-3 Extended Data Figure 4-3 Response accuracy for Pavlovian-congruent and conflict cards, plotted separately for Win and Avoid cards for block 1 (***A***) and block 2 (***B***). Please note that the concept of Pavlovian congruency cannot be interpreted in block 1 for the LowControl groups, as it just reflects the arbitrary labeling of the two Win cards as Go-to-Win and NoGo-to-Win, without underlying response-feedback contingency that could drive cue-response learning. Download Figure 4-3, TIF file.

10.1523/ENEURO.0041-21.2021.f4-4 Extended Data Figure 4-4 Percentage of Go responses (PercGo) for Pavlovian-congruent and conflict cards, plotted separately for Win and Avoid cards for block 1 (***A***) and block 2 (***B***). Please note that the concept of Pavlovian congruency cannot be interpreted in block 1 for the LowControl groups, as it just reflects the arbitrary labeling of the two Win cards as Go-to-Win and NoGo-to-Win, without underlying response-feedback contingency that could drive cue-response learning. Download Figure 4-4, TIF file.

When comparing changes in response accuracy between blocks using Cumming estimation plots, we found effect size estimates with the 95% CI excluding zero for both LowControl groups (HighControl-Sham: −0.27 [−0.75, 0.09], HighControl-Stimulated: −0.16 [−0.42, 0.06], LowControl-Sham: 0.55 [0.14, 0.94], LowControl-Stimulated: 1.17 [0.73, 1.61]; Extended Data Fig. 4-2). To verify that this effect was larger in the LowControl-Stimulated than in the LowControl-Sham group, we calculated block 2 minus block 1 difference scores, and estimated effect sizes for the stimulated versus sham HD-tDCS comparison, separately in the HighControl and LowControl conditions (Fig. 4*B*). This analysis revealed negligible effect for HD-tDCS in the HighControl groups (0.15 [−0.39, 0.65]), but a medium mean effect size in the LowControl-Stimulated versus LowControl-Sham comparison (0.63 [0.10, 1.06]), confirming results from rmANOVA (Fig. 4*A*). This effect was robust against removing one participant from the LowControl-Stimulated group, who was the only one in the whole sample producing accuracy scores of both <0.3 and >0.7 in the two blocks, resulting in an extremely large difference score (Cohen’s *d* for the comparison of LowControl groups after exclusion: 0.56 [0.003, 1.06]).

The preregistered rmANOVA also indicated a significant congruency × valence × group interaction (*F*_(3,99)_ = 3.49, *p *=* *0.019, η_p_^2^ = 0.096; Fig. 4*C*). Here, all groups responded more accurately to Pavlovian-congruent cards in the loss domain (NoGo-to-Avoid > Go-to-Avoid; *p_Bonferroni_* < 0.003), an effect that was consistent across both blocks (Extended Data Fig. 4-3). For rewarding cards, however, no clear effect of congruency was found (*p_Bonferroni_* > 0.099), except for the LowControl-Stimulated group, where performance was surprisingly worse for Pavlovian-congruent Go-to-Win versus conflicting NoGo-to-Win cards (*p_Bonferroni_* = 0.008). Consequently, accuracy was comparable between groups for all Avoid cards as well as in conflicting NoGo-to-Win trials (*p_Bonferroni_* > 0.074), but for Pavlovian-congruent Go-to-Win cards, the LowControl-Stimulated group’s performance was worse than that of HighControl-Stimulated (*p_Bonferroni_* = 0.009) and HighControl-Sham participants (*p_Bonferroni_* = 0.002). The paradoxical effect of improved responding to Pavlovian-conflict Win cards in LowControl participants was most pronounced in LowControl-Stimulated participants in both blocks (Extended Data Fig. 4-3). While in block 2, this was because of improved responding to conflict NoGo-to-Win cards, in block 1, the effect was driven by reduced accuracy for congruent Go-to-Win cards. Given that controllability manipulation in the first block invalidates the concept of Pavlovian congruency (out of the two Win and two Avoid cards, one is arbitrarily labeled as Go and one as NoGo, but these attributes cannot be learned in the absence of action-outcome contingency), the number of Go responses must necessarily be similar for congruent versus conflict cards in this block. To verify this, we also calculated the percentage of Go responses for each card type and block. Indeed, the main effect of Congruency was not significant for block 1 in LowControl groups either for Win or Avoid cards (*F *<* *1.43, *p *>* *0.237), while it was significant for HighControl groups in block 1 and all four groups in block 2, in both the gain and loss domains (all *F *>* *12.75, *p *<* *0.001; Extended Data Fig. 4-4).

Finally, the significant congruency × valence × block interaction for response accuracy (*F*_(1,99)_ = 8.89, *p *=* *0.004, η_p_^2^ = 0.082) was because of improved performance from block 1 to block 2 for NoGo cards only (NoGo-to-Avoid: *p_Bonferroni_* < 0.001, NoGo-to-Win: *p_Bonferroni_* = 0.036, Go-to-Avoid: *p_Bonf_* = 0.074, Go-to-Win: *p_Bonf_* = 0.055), albeit this effect was independent of group (four-way interaction: *F*_(3,99)_ = 0.29, *p *=* *0.833, η_p_^2^ = 0.009).

### Computational modeling

Modeling in this study was not preregistered, and thus, it should be regarded as exploratory. We extracted four latent parameters of learning and decision-making: PB (*π*), learning rate (*α*), temperature (*β*) and Go-bias (*b_go_*).

**Figure 5.**
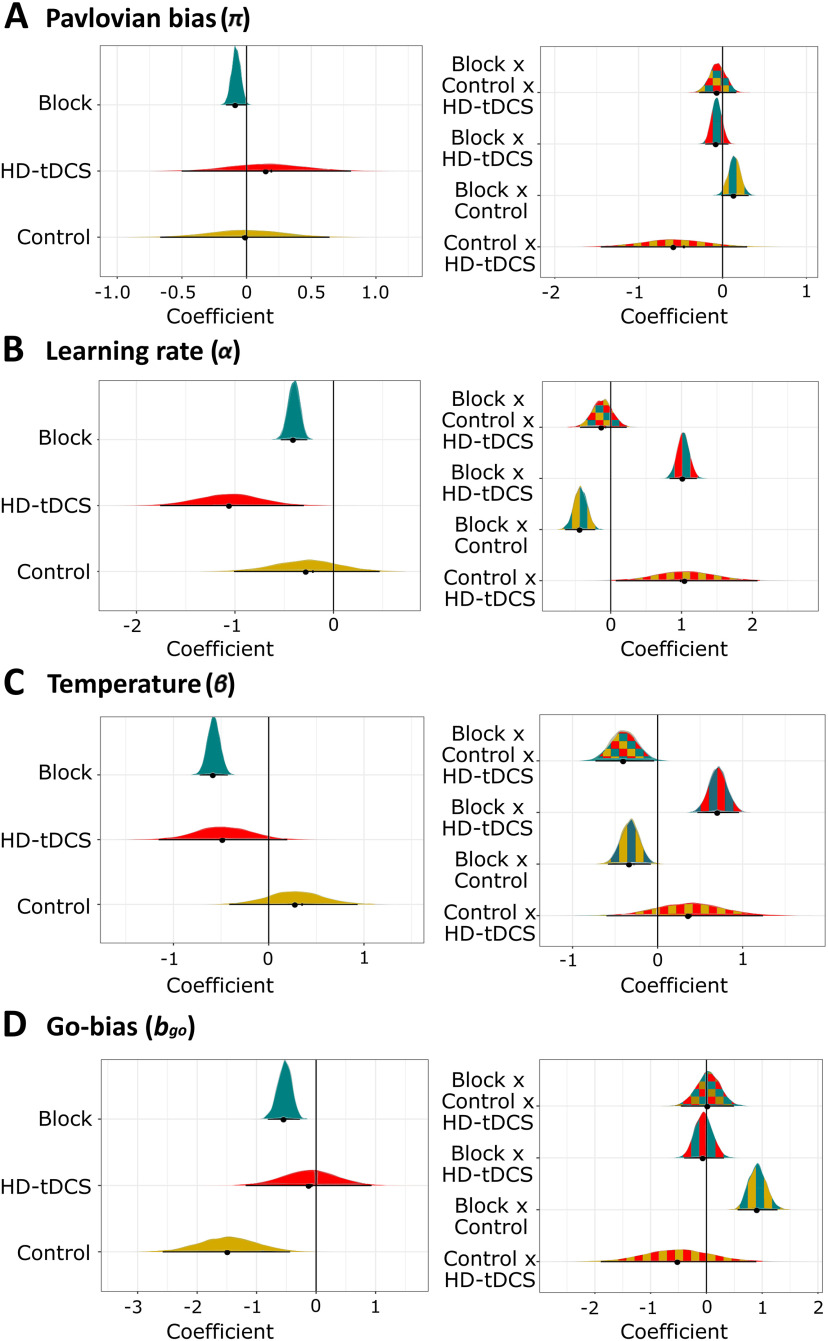
Results from computational modeling. Posterior densities for estimates for the regression coefficients for PB (parameter *π*; ***A***), learning rate (parameter *α*; ***B***), temperature/randomness of choice (parameter *β*; ***C***), and Go-bias (parameter *b_go_*; ***D***).

Posterior distributions for the group-level distribution of the Pavlovian parameter were in the positive range (*π* = 0.59, [0.20, 0.99]), confirming that learned stimulus valence biased decisions in the expected manner (action/inaction for positive/negative values, respectively). The estimated 95% HDI for the Block coefficient on the PB was negative (*b* = −0.08 [−0.15, −0.01], *P(b < 0) *=* *0.993, ER_–_ = 135.3), in line with results from rmANOVA that PB was partially learnt away by block 2. Moreover, predominantly positive values for the block × control interaction (0.14 [−0.01, 0.29], *P(b > 0) *=* *0.974, ER_+_ = 33.3; Fig. 5*A*) provided evidence for our hypothesis that controllability interfered with reductions in PB throughout the task (i.e., changes in PB from block 1 to block 2 were weaker in LowControl groups). Lastly, we found some support for the interaction between controllability and HD-tDCS (i.e., weaker PB in the LowControl-Stimulated group; *b* = −0.58 [−1.37, 0.17], *P(b < 0) *=* *0.927, ER_–_ = 12.7). Although the result indicated that posterior estimates for the control × HD-tDCS interaction were 12.7-times more likely to be in the negative range than being either positive or zero (in line with Fig. 3*B*,*C*), the effect was not compelling since the 95% HDI included zero.

Next, we looked at the learning rate parameter *α* resembling the degree to which participants updated their stimulus-action values on a trial-by-trial basis. Group-level coefficients were in a similar range as observed in previous reports (*α* = 0.20, [0.09, 0.34]). Again, we found negative values for the Block coefficient, indicating reduced learning rates by block 2 (*b* = −0.40 [−0.52, −0.28], *P(b < 0) *>* *0.999, ER_–_ = ∞; Fig. 5*B*). This effect was modulated by controllability and HD-tDCS in the opposite manner, with stronger reduction in LowControl participants (block × control: *b* = −0.43 [−0.62, −0.24], *P(b < 0) *>* *0.999, ER_–_ = ∞), but weaker change following real stimulation (block × HD-tDCS: *b *=* *1.03 [0.86, 1.20], *P(b > 0) *>* *0.999, ER_+_ = ∞). While the main effect of HD-tDCS was negative (*b* = −1.05 [−1.69, −0.43], *P(b < 0) *=* *0.999, ER_–_ = 749.0), positive values for the control × HD-tDCS interaction coefficient implied that stimulation of mPFC was less effective in reducing learning rates when combined with low controllability (*b *=* *1.05 [0.17, 1.93], *P(b > 0) *=* *0.991, ER_+_ = 100).

Posterior distributions for the temperature parameter (*β* = 2.71 [1.75, 3.99]) revealed a negative block-effect (*b* = −0.58 [−0.71, −0.45], *P(b < 0) *>* *0.999, ER_–_ = ∞; Fig. 5*C*), which can be interpreted as stronger reliance on learned action weights during response selection in block 2. Similarly to parameter *α*, low controllability intensified (block × control: *b* = −0.32 [−0.55, −0.11], *P(b < 0) *=* *0.998, ER_–_ = 544.4), while HD-tDCS attenuated this effect (block × HD-tDCS: *b *=* *0.71 [0.50, 0.91], *P(b > 0) *>* *0.999, ER_+_ = ∞). Importantly, participants acted less randomly in block 2 when low controllability was combined with HD-tDCS (block × control × HD-tDCS: b = −0.39 [−0.71, −0.10], *P(b < 0) *=* *0.994, ER_–_ = 170.4). We also found some support for a general reduction in *β* values in both groups with HD-tDCS, although the 95% HDI did not exclude zero (HD-tDCS: *b* = −0.48 [−1.07, 0.09], *P(b < 0) *=* *0.948, ER_–_ = 18.2).

The fourth parameter, $b_{go}$, represented Go-bias, the tendency to initiate actions regardless of learned stimulus value. As expected, value estimates were mostly positive ( $b_{go}$= 0.30, [−0.40, 0.99]), although associated with high uncertainty. The coefficient for block was negative (*b* = −0.53 [−0.78, −0.30], *P(b < 0) *>* *0.999, ER_–_ = ∞; Fig. 5*D*), indicating improved response accuracy from block 1 to block 2 in NoGo trials (see also Extended Data Fig. 4-1). We found strong evidence for the general reduction in Go-bias in LowControl groups (control: *b* = −1.48 [−2.35, −0.46], *P(b < 0) *=* *0.999, ER_–_ = 665.7), mirroring motor passivity in animal studies of LH (Maier and Seligman, 2016). Importantly, this effect was much stronger in block 1 (block × control: *b *=* *0.92 [0.61, 1.23], *P(b > 0) *>* *0.999, ER_+_ = ∞), pointing toward weak transfer from controllability manipulation to block 2, where control over outcomes was regained.

## Discussion

With respect to our first study aim, we expected to observe enhanced PB during and following low outcome controllability under sham stimulation. We postulated that such a result would (1) support earlier findings of similar nature (Dorfman and Gershman, 2019; Csifcsák et al., 2020); and (2) pinpoint our controllability manipulation protocol as a potent experimental intervention for inducing decision-making strategies resembling LH in healthy adults. However, we could only find partial support for this hypothesis: while preregistered analyses revealed comparable PPI values for the HighControl-Sham and LowControl-Sham groups, the PB parameter from the computational model was modulated in the expected direction (i.e., controllability manipulation attenuated the reduction of PB from block 1 to block 2). The discrepancy between results from the model-free PPI analysis and the model-derived Pavlovian parameter was also reported by another study (Csifcsák et al., 2020), indicating that the two measures differ in their sensitivities to changes in PB under low controllability. While PPI is an aggregated measure that simply reflects the propensity of initiating Go responses exclusively in appetitive trials and withholding actions specifically when facing aversive cues, the Pavlovian parameter takes into account the dynamic learning process, i.e., how card values are learnt from trial-to-trial, and the degree to which this valence-specific knowledge contributes to subsequent choices. Therefore, our study supports the view that the model-derived Pavlovian parameter is a more sensitive measure to manipulations of controllability in this experimental setting.

Low outcome controllability was also associated with stronger NoGo tendencies, indicated by a clearly reduced Go-bias parameter. Therefore, our findings do not resolve the controversy about whether experimental conditions resembling LH-induction influence Pavlovian response tendencies or lead to general behavioral passivity, since both mechanisms were implicated in the current study. Notably, weaker Go-bias during our controllability manipulation could also be because of the presence of the Go-cost in our task, since refraining from actions was the optimal strategy for LowControl participants in block 1 to maximize their earnings. Altogether, we cannot state that our protocol for manipulating outcome controllability is potent in inducing decision-making patterns resembling LH. This conclusion is also supported by the absence of a transfer effect regarding response accuracy from block 1 to block 2 (i.e., comparable values for HighControl vs LowControl groups in the final block), and by the similar levels of perceived controllability across groups.

Our controllability manipulation schedule was not optimal for several reasons. First, by keeping the Go-cost, participants could exert some level of control over outcomes, and it also led to imperfect matching in reward/loss magnitude between HighControl and LowControl groups. Second, reduced control over rewards and losses increased uncertainty around action consequences, and thus, controllability was confounded with predictability (Ligneul, 2021). In this regard, neither our protocol, nor those previously described in the literature (Dorfman and Gershman, 2019; Csifcsák et al., 2020), including the seminal animal studies (Maier and Seligman, 1976), offer a “clean method” of controllability manipulations, with appropriately accounting for changes in outcome predictability. However, a new experimental setting has been introduced recently, that enables manipulations of controllability without confounding it with uncertainty (Ligneul et al., 2020). The authors show that an information-theoretic measure, transfer entropy, can efficiently capture environmental controllability, being dynamically inferred by the agent during the task. Future work could test whether other controllability manipulation schedules (similar to the one described by Ligneul et al., 2020) are more effective in inducing LH-like choice behavior in healthy adults.

The second aim of our study was to investigate how HD-tDCS above the mPFC influenced task performance under controllable versus largely uncontrollable response-outcome relationships. Our most striking result is that following low controllability, improvement in response accuracy was stronger in participants receiving HD-tDCS relative to those undergoing sham stimulation. This implies that stimulation above the mPFC/dACC led to more efficient adjustments in decision-making strategies following low controllability, when control over rewards and losses was regained. Although our simulations indicate that HD-tDCS-induced electric fields might have reached the dACC, these modeling results have not been validated by intracranial recordings, and therefore, they should be interpreted with caution. Nevertheless, the mPFC (and dACC in particular) has been associated with conditions of low controllability in the context of LH (Bauer et al., 2003; Diener et al., 2010), but also with tracking changes in environmental volatility (Behrens et al., 2007) and the implementation of CC during various cognitive tasks (Shenhav et al., 2013; Cavanagh and Frank, 2014). Interestingly, a recent study found evidence for trial-by-trial correspondence between frontal midline theta power (an electrophysiological correlate of dACC activity, associated with the implementation of CC), and subjectively inferred controllability in a very similar Go/NoGo task (Gershman et al., 2021). Moreover, anodal tDCS above the rostromedial PFC enhanced gathering information about one’s sense of being in control in a social context (Ligneul et al., 2016). Based on these findings, a possible mechanism for the observed effect in our study is that HD-tDCS improved the precision of controllability estimations rather than affecting PB per se. Following that interpretation, the LowControl-Stimulated group could more effectively adjust decision-making strategies to environmental constraints in both blocks. This is apparent in their lower number of Go responses for Win cards in the first block, a behavior that has been associated with reduced exploration tendencies (Teodorescu and Erev, 2014). Withholding Go tendencies was adaptive in this context, since active responses reduced the magnitude of outcomes by the Go-cost, without directly influencing reward/loss frequency. Conversely, in block 2, the LowControl-Stimulated group adjusted their choices in Pavlovian-conflict trials only, a response pattern that has been associated with increased cognitive effort (Cavanagh et al., 2013; Swart et al., 2018). Thus, we conclude that HD-tDCS above the mPFC facilitated LowControl participants’ task performance in an adaptive way, possibly via improving their assessment of environmental controllability.

The interaction between controllability and HD-tDCS was most striking in Win trials, resulting in better accuracy for conflicting NoGo-to-Win versus congruent Go-to-Win cards in the LowControl-Stimulated group. Moreover, this group was the only one to show improved responding to Go-to-Win cards from block 1 to block 2. These observations suggest that only LowControl-Stimulated participants could successfully suppress maladaptive PB in conflicting NoGo-to-Win trials by the end of the task, without overcompensating this strategy at the expense of congruent Go-to-Win cards. Such a selective overcompensation of NoGo response tendencies in Win trials (resulting in a reversal of the congruency effect) was also reported in another study, albeit only for participants with higher levels of outcome controllability (Csifcsák et al., 2020). A possible reason for this discrepancy is that LowControl participants in the study by Csifcsák et al. (2020) were initially exposed to a controllable version of the task, whereas in the current task, they immediately started the task with the manipulated block. Therefore, we propose that prior exposure to controllable outcomes plays a key role in how uncontrollable task-contexts shape decision-making. It remains puzzling, however, why we found stronger HD-tDCS effects for reward-predictive trials. While explanation for this phenomenon awaits future investigation, it is noteworthy that a recent neuroimaging study reported valence-action mappings during the processing of preparatory cues in the ACC for win (but not loss) trials, as well as increased ACC activity during target stimulus presentation for both approach versus avoid and win versus loss stimuli (Hoofs et al., 2021). Therefore, we speculate that this region is more sensitive to evolutionary dominant action requirements in the appetitive domain.

Recent studies suggest that PB during reinforcement learning is regulated by top-down CC mechanisms (Cavanagh et al., 2013; Swart et al., 2018; Csifcsák et al., 2020). An alternative explanation for the observed effect concerning the interaction between HD-tDCS and controllability is that, rather than influencing estimations of one’s controllability over the environment, mPFC stimulation directly facilitated the implementation of CC in Pavlovian-conflict trials. Conflict-associated CC has been successfully modulated via transcranial electric stimulation above midline frontal areas in a Stroop task (To et al., 2018), and in a similar orthogonalized Go/NoGo task (Turi et al., 2020). Thus, it is feasible that the combined effect of low controllability and HD-tDCS on response accuracy in our study is related to enhanced CC and the consequential suppression of PB. In line with this argument, we found improved accuracies for both NoGo-to-Win and Go-to-Avoid cards in block 2 for LowControl-Stimulated versus LowControl-Sham participants. Moreover, Cumming estimation plots provided some evidence for weaker PB in the LowControl-Stimulated group, predominantly in block 1. However, it should be noted that results from the preregistered PPI analysis and the computational modeling approach were not compelling, so this conclusion based on exploratory analyses should be treated with caution.

One could argue that instead of reducing PB for Win cards, HD-tDCS might have promoted NoGo tendencies globally in LowControl-Stimulated participants. While the model-derived Go-bias parameter is inconclusive for the control × HD-tDCS interaction, our results from PPI and accuracy analyses argue against this interpretation. First, a possible general facilitation of NoGo responding should have influenced the two PB sub-measures (reward-based invigoration and punishment-based suppression) in the opposite direction, but this effect was not present in our results. More specifically, the predisposition for NoGo responses in the LowControl-Stimulated group was not present in Avoid trials (producing even more Go responses in Go-to-Avoid trials in block 2, compared with LowControl-Sham participants) which points toward the specificity of NoGo-enhancement to Win trials. In line with the PB account, Ly and colleagues have reported reduced “affective biasing” of instrumental responding following cathodal tDCS above the frontopolar cortex (Ly et al., 2016). In that study, approach primed by emotional stimuli (roughly equivalent to our Go trials) was faster for appetitive versus aversive cues during sham stimulation, but this effect was reversed in the cathodal condition. Given the diffuse cortical distribution of tDCS-induced electric fields in that protocol (with the return electrode placed above the occipital area), it is possible that the similar finding of weaker PB following tDCS in the two studies was mediated by excitability changes in overlapping cortical areas.

The fact that HD-tDCS did not influence response accuracy in HighControl participants is a surprising finding, as we expected that the HighControl-Stimulated group would show more efficient CC and improved task performance in Pavlovian-conflict trials. It is possible that our controllability manipulation changed neural excitability in target regions so that they became more susceptible to HD-tDCS-induced electric fields. Indeed, behavioral consequences of tDCS are sensitive to dynamic fluctuations in neural activity during stimulation (“state dependency”; Tremblay et al., 2014; Dubreuil-Vall et al., 2019), and a recent study implementing a similar low controllability protocol reported altered neurophysiological responses arising from the mPFC/dACC in LowControl subjects (Csifcsák et al., 2020). Based on these results, we speculate that HD-tDCS in our study was not potent enough to influence task performance in participants with sufficient levels of outcome controllability, whereas controllability manipulation might have lowered the threshold for HD-tDCS effects to develop.

Computational modeling revealed two additional latent processes that were influenced by our interventions. While earlier work reported stronger reliance on immediate feedback (i.e., increased learning rates) under reduced outcome controllability (Csifcsák et al., 2020), we found such an effect only when controllability was combined with HD-tDCS. Similarly, there was no conclusive evidence for increased exploration/randomness during choice selection in the LowControl groups, which is in contrast with previous findings (Csifcsák et al., 2020). However, controllability and HD-tDCS exerted opposite (intensifying and attenuating) effects on the reduction of both parameters from block 1 to block 2. Although we hesitate to provide an interpretation for these interactions as they stem from an exploratory analysis, the results suggest that evaluation of outcome controllability converges with both the rate of feedback learning and randomness of choice in the mPFC/dACC.

Our key finding is that HD-tDCS facilitated task performance in the LowControl group only, an effect that was stronger in the appetitive domain, and possibly related to more precise estimations of controllability and/or to enhanced CC over Pavlovian response tendencies. Moreover, controllability and HD-tDCS showed interactive effects in the gradual accumulation of stimulus-action values and in the tendency to act randomly rather than to rely on reinforcement history. Overall, these results highlight the potential of our protocol for interfering with choice arbitration under low controllability of environmental events, resulting in more adaptive behavior.

## Synthesis

Reviewing Editor: Christina Zelano, Northwestern University Decisions are customarily a result of the Reviewing Editor and the peer reviewers coming together and discussing their recommendations until a consensus is reached. When revisions are invited, a fact-based synthesis statement explaining their decision and outlining what is needed to prepare a revision will be listed below. The following reviewer(s) agreed to reveal their identity: Romain Ligneul, Verena Ly.

The reviewers agreed that this study on controllability beyond stress protocol is a timely topic which has a transformative potential for the study of learning and decision-making algorithms in the brain. The study is innovative and reports several interesting results. Both reviewers raised several specific and detailed concerns which are meant to improve the manuscript. See below their detailed comments. Please make sure to address all major concerns, paying particular attention to questions and concerns about statistics.

Reviewer 1:

Major concerns:

1) Was the randomization of outcomes performed in the yoked group valence-specific (i.e done separately for win and avoid cards) or not? Unless I am mistaking, this crucial information is missing. In the rest of this review, I will assume that it was valence-specific, based on the above-chance levels of PPI observed in block 1 for Yoked-Sham participants, which seems hard to explain otherwise.

2) Behaviorally, the complete disappearance of Pavlovian biases in the Yoked-Active condition as compared to all other conditions is the most striking result (Fig3A). The statistical power is not sufficient to establish this quantitatively through preregistered analyses, but the effect is qualitatively salient. I believe that this effect should be interpreted further. Indeed, the cognitive control hypothesis mentionned lines 414-420 does not explain why the reduction of PB seen in the Yoked-Active condition (and to a lesser extent, in the Yoked-Sham condition) would be circumscribed to the gain domain. What further complicates the interpretation is that this paradoxical result was not observed in a previous study, presumably from the same group (Csifcsák et al., 2020). The main difference between the two paradigms is that yoked participants never experienced controllable outcome before block 2 in the current study while they did in the previous one. Consequently, the present finding could reflect an actual effect of uncontrollable outcomes, masked in the previous study by prior exposure to controllable outcomes (which is known to be protective with respect to learned helplessness). In any case, it would be good unpack the results of Fig. 4C for each block in a supplementary Figure to allow readers to fully assess this finding.

3) The strong reduction of the Go-bias seen for Yoked participants (as shown by Fig. 5D) partly explains the results reported Fig. 4C. However, rather than suggesting that Yoking promoted a generalized no-go strategy opposite to what Guitart-Masip and colleagues reported for citalopram (2014), the authors try to frame their effect in terms of Pavlovian bias, which makes the whole slightly hard to understand. Why not simply acknowledging that Yoking promotes behavioral passivity? It is actually quite in line with the learned helplessness literature. Less with Dorfman and Gershman paper (Nat Comms, 2020), but in their study, the manipulation of controllability is confounded with a manipulation of outcome distributions and with the overall frequency of correct go responses, which implies that it should not be constrain any hypothesis or interpretation related to controllability. Instead, it would be important to emphasize the discrepancy observed with their study while insisting on the fact that the yoking protocol provides a “cleaner” method to manipulate controllability.

4) Yoking is cleaner, but it remains imperfect because it still confounds the controllability of rewards with the predictability of rewards, which is not a fatality. Indeed, while actions predict rewards in controllable environments, past states (e.g a cue) can predict rewards in uncontrollable environments. This confounds -or, say, this imperfection of the definition of Maier and Seligman (1976)- should be briefly acknowledged.

5) The authors focused their attention on the hypothesis that mPFC stimulation may influence the impact of outcome controllability on Pavlovian biases. However, the data does not support this primary effect, quite the opposite, and the interpretation of preregistered analyses regarding tDCS effects is therefore complicated. In my view, what is missing in the manuscript is the possibility that mPFC stimulation acted on controllability estimation per se, and more precisely, helped participants to switch between controllability conditions. In the first block, Yoked-Active invested less effort than Yoked-Sham, while they invested more in the second block, which is exactly what would be expected under this alternative hypothesis. Controllability estimation is in itself a complicated process, but a recent study has shown that transfer entropy can be used to quantify the amount of evidence that exploration generates to determine whether an environment is controllable or not. I encourage the authors to compute and report the mean transfer entropy for the 4 groups and two blocks independently. Indeed, one possibility is that the paradoxical results seen for Win cards reflect an exploratory strategy through which the Yoked groups kept evaluation their level of control on outcomes throughout the task. If this is the case, transfer entropy should be higher for Yoked than Control participants in the second block (in the first block, it will be lower in Yoked participants by design, but it might still be interesting to see interindividual variability in TE, since chance can lead to spuriously high TE values. In the case of authors: TE=H(Outcome|Card)-H(Outcome|Card,Action).

https://www.biorxiv.org/content/10.1101/2020.11.19.390393v2

6) Potentially, TE could be assessed separately for each valence.

Minor points:

a. It might be useful to relate the current results with those previously obtained in cognitive tasks using mPFC-tDCS. For example, Ligneul and colleagues (Current Biology 2016) had found that anodal mPFC-tDCS could modulate learning rates in a competitive perceptual decision making task. They also reported an increased temporal autocorrelation of choices (which could be interesting to look at in the current study too). While these effects were interpreted in the context of social dominance learning, it is worth to note that social dominance is defined by the existence of asymmetries of control over resources and others’ behaviors, and that subordination can lead to learned helplessness too.

b. Regarding the mPFC-DRN relationship, I agree that there is a general consensus that mPFC projection play a causal role in the protective effect of control. Given that the main effect of Yoking in the current study is to induce a reduction of the Go-bias, an effect partly (albeit non significantly?) alleviated by tDCS, it may be worth citing the paper by Warden et al. (Nature, 2012) showing that the stimulation of mPFC projections within the DRN increases locomotor activity in the forced-swim test.

c. I think that it is misleading to refer to studies which manipulated the uncertainty or volatility of the environment as controllability studies (e.g. Behrens at line 439, Dorfman and Gershman, etc.).

d. Given that the spatial extent of the HD-tDCS protocol is based on simulations which may not be completely consensual in the field, it might be worth being slightly more cautious regarding the claim that dACC was actually modulated.

e. I think that the abstract should mention the unexpected effect of the yoking procedure in appetitive trials.

f. The a priori power analysis should be detailed further. What data was used to compute it, what is the target test?

g. Perhaps reaction times could be analyzed in order interpret further the effects of Yoking and tDCS. For example, is the reduction of go-bias associated with an increase of reaction times or not? If it is, then it would suggest the lack of control can promote a form of low level behavioral inhibition. If it is not, then the paradoxical choice pattern most likely derives from a higher level impact on decision-making strategies.

Conclusion:

The unexpected findings of these study, albeit challenging for preregistered analyses, constitute in my view an important addition to the field. It would be useful to replicate but this is beyond the scope of the current paper. If the raw data pertaining to choice and RTs is published online, I am confident that it will lead to reanalyses and that it will constitute a useful basis for updated hypotheses regarding the influence of Yoking and/or the modulation of learning and decision-making by mPFC-tDCS.

Reviewing this paper was also interesting because it constitutes an interesting example of the conflict which can exist between new practices in science: double-blind review on the one hand and preregistration on the other hand. If the reviewer cannot access any preregistered information, it is impossible to evaluate an important part of the manuscript. It also shows that blinding the reviewer is complicated when it comes to studies which are heavily based on another previously published study. Overall, I feel that double-blinding was counter-productive in this case: while I have little doubts about the group who authored the study, I could not access the code, raw data or preregistration, hence limiting my capacity to provide informative feedback.

Reviewer 2

The authors aimed to test if low control would increase a Pavlovian bias and if HD-tDCS above the mPFC would reduce this effect. Although this is an interesting study and potentially could provide advances in the field, there are some major concerns and limitations.

Major concerns:

- The task (design) is not explained sufficiently. How did the Yoking condition look like exactly? That is, how was the randomization done with regards to the outcomes that were yoked to the matched participant? This information is needed to be able to fully evaluate the validity of the design and to understand the manipulation. Since there was no sufficient evidence for an effect of the manipulation on subjective succes or controllability and the main effect of the task - Pavlovian bias effect (particularly in the win condition), it is important to consider the details of the task thoroughly.Furthermore, what were the instructions that the participants received for the task? Was it clear to the participants there were win vs lose cards? ‘Yoking’ is not a term that technically equals low controllability, but is rather used to indicate a control condition yoked to an experimental condition. It may therefore be confusing for the reader to use the term for an experimental condition and use ‘control’ for the condition with high reward probability. It would be better to choose other labels for the condition and/or explain them more clearly.

- The authors describe the environment as volatile (e.g. in abstract), what do the authors refer to with this? Their manipulation of yoked vs control condition is not merely affecting the controllability indeed, but also the volatility, and also uncertainty; and depending on how the outcomes were randomized in the yoked condition, also the reward frequency/rates.

- what were the precise hypothesis with regard to the interaction between the block and stimulation? The stimulation may have online and after effects, but the online effects may interact with the manipulation during block 1. This could be clarified.

- It would have made sense to run a 2x2x2 mixed design ANOVA given the design. The rationale for the exploratory analyses Cumming plots and computational modeling is missing. I also wonder whether or not the computational model is optimal for these data. Model comparisons with simpler models would be needed to demonstrate this.

- I wonder whether the findings on accuracy are robust, using these statistics; it looks like the effect could be driven by some participants who show either a floor or ceiling effect in the first or second block (difference in accuracy of .6).

-Although I appreciate that the authors report exploratory analyses on top of the preregistered analyses, it is difficult to reconcile the findings on the different parameters of the computational model with the model-free findings. One reason is the lack of a clear rationale for the type of exploratory analyses. The authors could discuss more how the parameters from computational model could be informative with regards to the study aims and how they relate to the model-free findings.

-It may be helpful to look into the response latency effects as well; Pavlovian bias may be reflected by reaction time

- What is on the Y-axis in Fig 4C? I am surprised by the lack of a bias effect in the win condition and that it is opposite even for the yoked condition.

## References

[B1] Amat J, Baratta MV, Paul E, Bland ST, Watkins LR, Maier SF (2005) Medial prefrontal cortex determines how stressor controllability affects behavior and dorsal raphe nucleus. Nat Neurosci 8:365–371. 10.1038/nn1399 15696163

[B2] Bauer H, Pripfl J, Lamm C, Prainsack C, Taylor N (2003) Functional neuroanatomy of learned helplessness. Neuroimage 20:927–939. 10.1016/S1053-8119(03)00363-X 14568463

[B3] Beck AT, Weissman A, Lester D, Trexler L (1974) The measurement of pessimism: the hopelessness scale. J Consult Clin Psychol 42:861–865. 10.1037/h0037562 4436473

[B4] Behrens TE, Woolrich MW, Walton ME, Rushworth MF (2007) Learning the value of information in an uncertain world. Nat Neurosci 10:1214–1221. 10.1038/nn1954 17676057

[B5] Boayue NM, Csifcsák G, Puonti O, Thielscher A, Mittner M (2018) Head models of healthy and depressed adults for simulating the effects of non-invasive brain stimulation [version 2; referees: 2 approved]. F1000Res 7:704. 10.12688/f1000research.15125.2 30505431 PMC6241565

[B6] Carpenter B, Gelman A, Hoffman MD, Lee D, Goodrich B, Betancourt M, Brubaker M, Guo J, Li P, Riddell A (2017) Stan: a probabilistic programming language. J Stat Softw 76:1–32. 10.18637/jss.v076.i01 PMC9788645 36568334

[B7] Carver CS, White TL (1994) Behavioral inhibition, behavioral activation, and affective responses to impending reward and punishment: the BIS/BAS scales. J Pers Soc Psychol 67:319–333. 10.1037/0022-3514.67.2.319

[B8] Cavanagh JF, Frank MJ (2014) Frontal theta as a mechanism for cognitive control. Trends Cogn Sci 18:414–421. 10.1016/j.tics.2014.04.012 24835663 PMC4112145

[B9] Cavanagh JF, Eisenberg I, Guitart-Masip M, Huys Q, Frank MJ (2013) Frontal theta overrides pavlovian learning biases. J Neurosci 33:8541–8548. 10.1523/JNEUROSCI.5754-12.2013 23658191 PMC3707146

[B10] Csifcsák G, Boayue NM, Puonti O, Thielscher A, Mittner M (2018) Effects of transcranial direct current stimulation for treating depression: a modeling study. J Affect Disord 234:164–173. 10.1016/j.jad.2018.02.077 29529550

[B11] Csifcsák G, Melsæter E, Mittner M (2020) Intermittent absence of control during reinforcement learning interferes with Pavlovian bias in action selection. J Cogn Neurosci 32:646–663. 10.1162/jocn_a_01515 31851595

[B12] Datta A, Bansal V, Diaz J, Patel J, Reato D, Bikson M (2009) Gyri-precise head model of transcranial direct current stimulation: improved spatial focality using a ring electrode versus conventional rectangular pad. Brain Stimul 2:201–207. 10.1016/j.brs.2009.03.005 20648973 PMC2790295

[B13] Dayan P, Berridge KC (2014) Model-based and model-free Pavlovian reward learning: revaluation, revision, and revelation. Cogn Affect Behav Neurosci 14:473–492. 10.3758/s13415-014-0277-8 24647659 PMC4074442

[B14] Dayan P, Niv Y, Seymour B, Daw ND (2006) The misbehavior of value and the discipline of the will. Neural Netw 19:1153–1160. 10.1016/j.neunet.2006.03.002 16938432

[B15] Diener C, Kuehner C, Flor H (2010) Loss of control during instrumental learning: a source localization study. Neuroimage 50:717–726. 10.1016/j.neuroimage.2009.12.094 20045474

[B16] Dorfman HM, Gershman SJ (2019) Controllability governs the balance between Pavlovian and instrumental action selection. Nat Commun 10:5826–5828. 10.1038/s41467-019-13737-7 31862876 PMC6925275

[B17] Dubreuil-Vall L, Chau P, Ruffini G, Widge AS, Camprodon JA (2019) tDCS to the left DLPFC modulates cognitive and physiological correlates of executive function in a state-dependent manner. Brain Stimul 12:1456–1463. 10.1016/j.brs.2019.06.006 31221553 PMC6851462

[B18] Gelman A, Rubin DB (1992) Inference from iterative simulation using multiple sequences. Statist Sci 7:457–472. 10.1214/ss/1177011136

[B19] Gelman A, Carlin JB, Stern HS, Rubin DB (2003) Bayesian data analysis. London: Chapman and Hall/CRC Texts in Statistical Science.

[B20] Gershman SJ, Guitart-Masip M, Cavanagh JF (2021) Neural signatures of arbitration between Pavlovian and instrumental action selection. PLoS Comput Biol 17:e1008553. 10.1371/journal.pcbi.1008553 33566831 PMC7901778

[B21] Guitart-Masip M, Huys QJ, Fuentemilla L, Dayan P, Duzel E, Dolan RJ (2012) Go and no-go learning in reward and punishment: interactions between affect and effect. Neuroimage 62:154–166. 10.1016/j.neuroimage.2012.04.024 22548809 PMC3387384

[B22] Hershberger WA (1986) An approach through the looking-glass. Anim Learn Behav 14:443–451. 10.3758/BF03200092

[B23] Ho J, Tumkaya T, Aryal S, Choi H, Claridge-Chang A (2019) Moving beyond P values: data analysis with estimation graphics. Nat Methods 16:565–566. 10.1038/s41592-019-0470-3 31217592

[B24] Hoffman MD, Gelman A (2014) The No-U-turn sampler: adaptively setting path lengths in Hamiltonian Monte Carlo. J Mach Learn Res 15:1593–1623.

[B25] Hoofs V, Park HR, Vermeylen L, Boehler CN, Krebs RM (2021) Neural underpinnings of valence-action interactions triggered by cues and targets in a rewarded approach/avoidance task. Cortex 141:240–261. 10.1016/j.cortex.2021.04.013 34098425

[B26] Huys QJM, Eshel N, O'Nions E, Sheridan L, Dayan P, Roiser JP (2012) Bonsai trees in your head: how the Pavlovian system sculpts goal-directed choices by pruning decision trees. PLoS Comput Biol 8:e1002410. 10.1371/journal.pcbi.1002410 22412360 PMC3297555

[B27] Kerr DL, McLaren DG, Mathy RM, Nitschke JB (2012) Controllability modulates the anticipatory response in the human ventromedial prefrontal cortex. Front Psychol 3:557. 10.3389/fpsyg.2012.00557 23550176 PMC3582324

[B28] LeDoux J, Daw ND (2018) Surviving threats: neural circuit and computational implications of a new taxonomy of defensive behaviour. Nat Rev Neurosci 19:269–282. 10.1038/nrn.2018.22 29593300

[B29] Ligneul R (2021) Prediction or causation? Towards a redefinition of task controllability. Trends Cogn Sci 25:431–433. 10.1016/j.tics.2021.02.009 33712402

[B30] Ligneul R, Obeso I, Ruff CC, Dreher JC (2016) Dynamical representation of dominance relationships in the human rostromedial prefrontal cortex. Curr Biol 26:3107–3115. 10.1016/j.cub.2016.09.015 28094034

[B31] Ligneul R, Mainen Z, Ly V, Cools R (2020) Stress-sensitive inference of task controllability. bioRxiv 2020.11.19.390393. 10.1038/s41562-022-01306-w 35273354

[B32] Ly V, Bergmann TO, Gladwin TE, Volman I, Usberti N, Cools R, Roelofs K (2016) Reduced affective biasing of instrumental action with tDCS over the prefrontal cortex. Brain Stimul 9:380–387. 10.1016/j.brs.2016.02.002 26968807 PMC4881415

[B33] Ly V, Wang KS, Bhanji J, Delgado MR (2019) A reward-based framework of perceived control. Front Neurosci 13:65. 10.3389/fnins.2019.00065 30809112 PMC6379460

[B34] Maier SF, Seligman ME (1976) Learned helplessness: theory and evidence. J Exp Psychol Gen 105:3–46. 10.1037/0096-3445.105.1.3

[B35] Maier SF, Seligman MEP (2016) Learned helplessness at fifty: insights from neuroscience. Psychol Rev 123:349–367. 10.1037/rev0000033 27337390 PMC4920136

[B36] Maier SF, Watkins LR (2005) Stressor controllability and learned helplessness: the roles of the dorsal raphe nucleus, serotonin, and corticotropin-releasing factor. Neurosci Biobehav Rev 29:829–841. 10.1016/j.neubiorev.2005.03.021 15893820

[B37] Peirce JW (2007) PsychoPy—psychophysics software in Python. J Neurosci Methods 162:8–13. 10.1016/j.jneumeth.2006.11.017 17254636 PMC2018741

[B38] Pryce CR, Azzinnari D, Spinelli S, Seifritz E, Tegethoff M, Meinlschmidt G (2011) Helplessness: a systematic translational review of theory and evidence for its relevance to understanding and treating depression. Pharmacol Ther 132:242–267. 10.1016/j.pharmthera.2011.06.006 21835197

[B39] Rahman A, Reato D, Arlotti M, Gasca F, Datta A, Parra LC, Bikson M (2013) Cellular effects of acute direct current stimulation: somatic and synaptic terminal effects. J Physiol 591:2563–2578. 10.1113/jphysiol.2012.247171 23478132 PMC3678043

[B40] Rangel A, Camerer C, Montague PR (2008) A framework for studying the neurobiology of value-based decision making. Nat Rev Neurosci 9:545–556. 10.1038/nrn2357 18545266 PMC4332708

[B41] Shenhav A, Botvinick MM, Cohen JD (2013) The expected value of control: an integrative theory of anterior cingulate cortex function. Neuron 79:217–240. 10.1016/j.neuron.2013.07.007 23889930 PMC3767969

[B42] Swart JC, Frank MJ, Määttä JI, Jensen O, Cools R, den Ouden HE (2018) Frontal network dynamics reflect neurocomputational mechanisms for reducing maladaptive biases in motivated action. PLoS Biol 16:e2005979. 10.1371/journal.pbio.2005979 30335745 PMC6207318

[B43] Teodorescu K, Erev I (2014) Learned helplessness and learned prevalence: exploring the causal relations among perceived controllability, reward prevalence, and exploration. Psychol Sci 25:1861–1869. 10.1177/0956797614543022 25193942

[B44] Thielscher A, Antunes A, Saturnino GB (2015) Field modeling for transcranial magnetic stimulation: a useful tool to understand the physiological effects of TMS? Annu Int Conf IEEE Eng Med Biol Soc 2015:222–225. 26736240 10.1109/EMBC.2015.7318340

[B45] To WT, Eroh J, Hart J, Vanneste S (2018) Exploring the effects of anodal and cathodal high definition transcranial direct current stimulation targeting the dorsal anterior cingulate cortex. Sci Rep 8:4454–4416. 10.1038/s41598-018-22730-x 29535340 PMC5849683

[B46] Tremblay S, Lepage JF, Latulipe-Loiselle A, Fregni F, Pascual-Leone A, Théoret H (2014) The uncertain outcome of prefrontal tDCS. Brain Stimul 7:773–783. 10.1016/j.brs.2014.10.003 25456566 PMC4342747

[B47] Turi Z, Mittner M, Lehr A, Bürger H, Antal A, Paulus W (2020) Theta-gamma cross-frequency transcranial alternating current stimulation over the trough impairs cognitive control. eNeuro 7:ENEURO.0126-20.2020. 10.1523/ENEURO.0126-20.2020 PMC7540931 32764077

[B48] Turner ML, Engle RW (1989) Is working memory capacity task dependent? J Mem Lang 28:127–154. 10.1016/0749-596X(89)90040-5

[B49] Watson D, Clark LA, Tellegen A (1988) Development and validation of brief measures of positive and negative affect: the PANAS scales. J Pers Soc Psychol 54:1063–1070. 10.1037/0022-3514.54.6.1063 3397865

