# Supplementary figures and images for: Transcranial Direct Current Stimulation above the Medial Prefrontal Cortex Facilitates Decision-Making following Periods of Low Outcome Controllability
Source: eNeuro. 2021 Sep 7;8(5):ENEURO.0041-21.2021. doi: 10.1523/ENEURO.0041-21.2021 (PMC8425969; doi:10.1523/ENEURO.0041-21.2021)

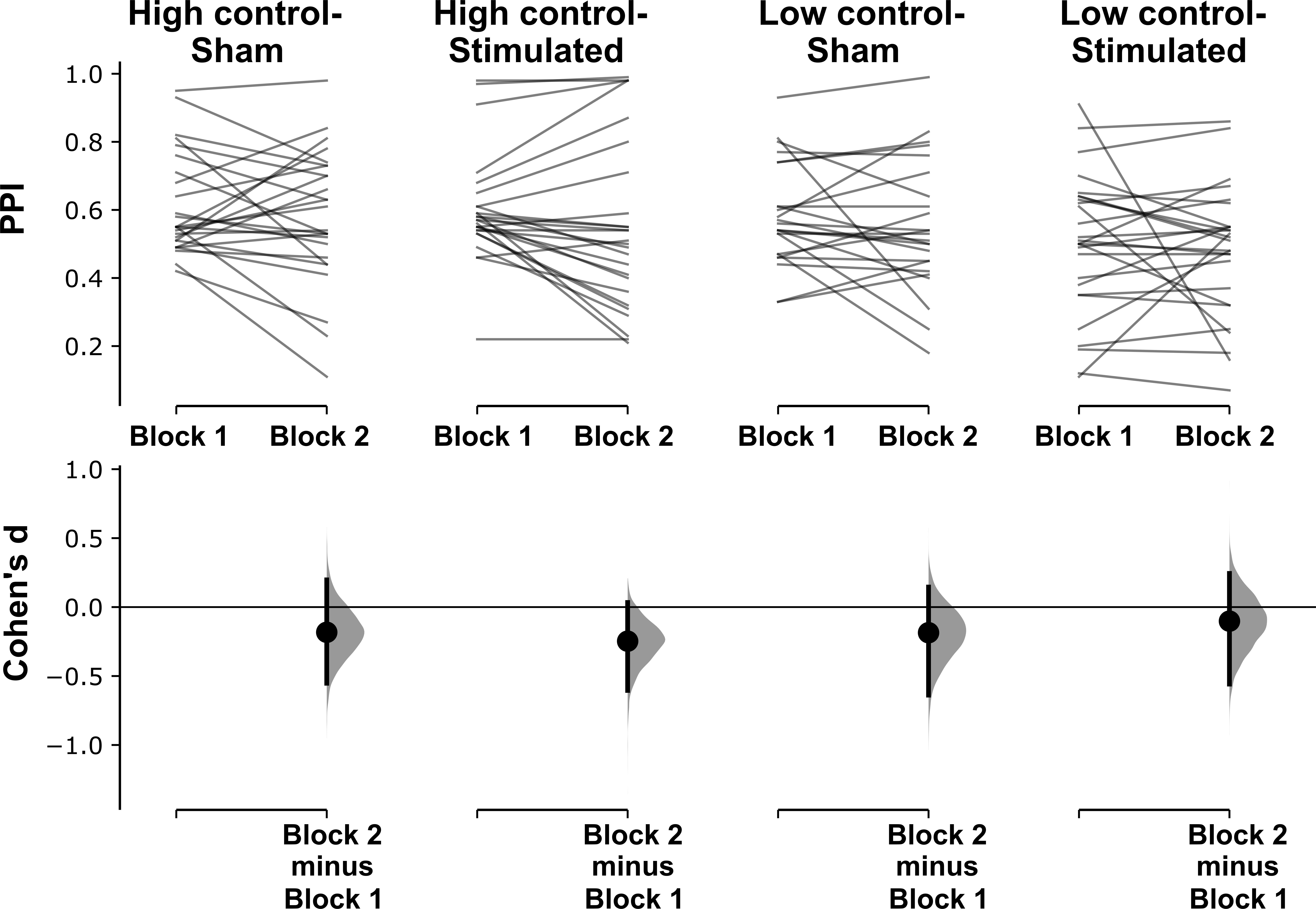

Supplement: Extended Data Figure 3-1 — Raw data (upper panel) showing changes in the PPI from block 1 to block 2 for each participant, and Cumming estimation plots (lower panel) representing effect size estimates (Cohen’s d) for the change in response accuracy from block 1 to block 2, plotted separately for the four groups. Mean differences are presented as dots, along with the corresponding bootstrap sampling distributions (5000 samples) and the bias-corrected and accelerated 95% CIs. Download Figure 3-1, TIF file. [file enu-eN-NWR-0041-21-s02.tif]

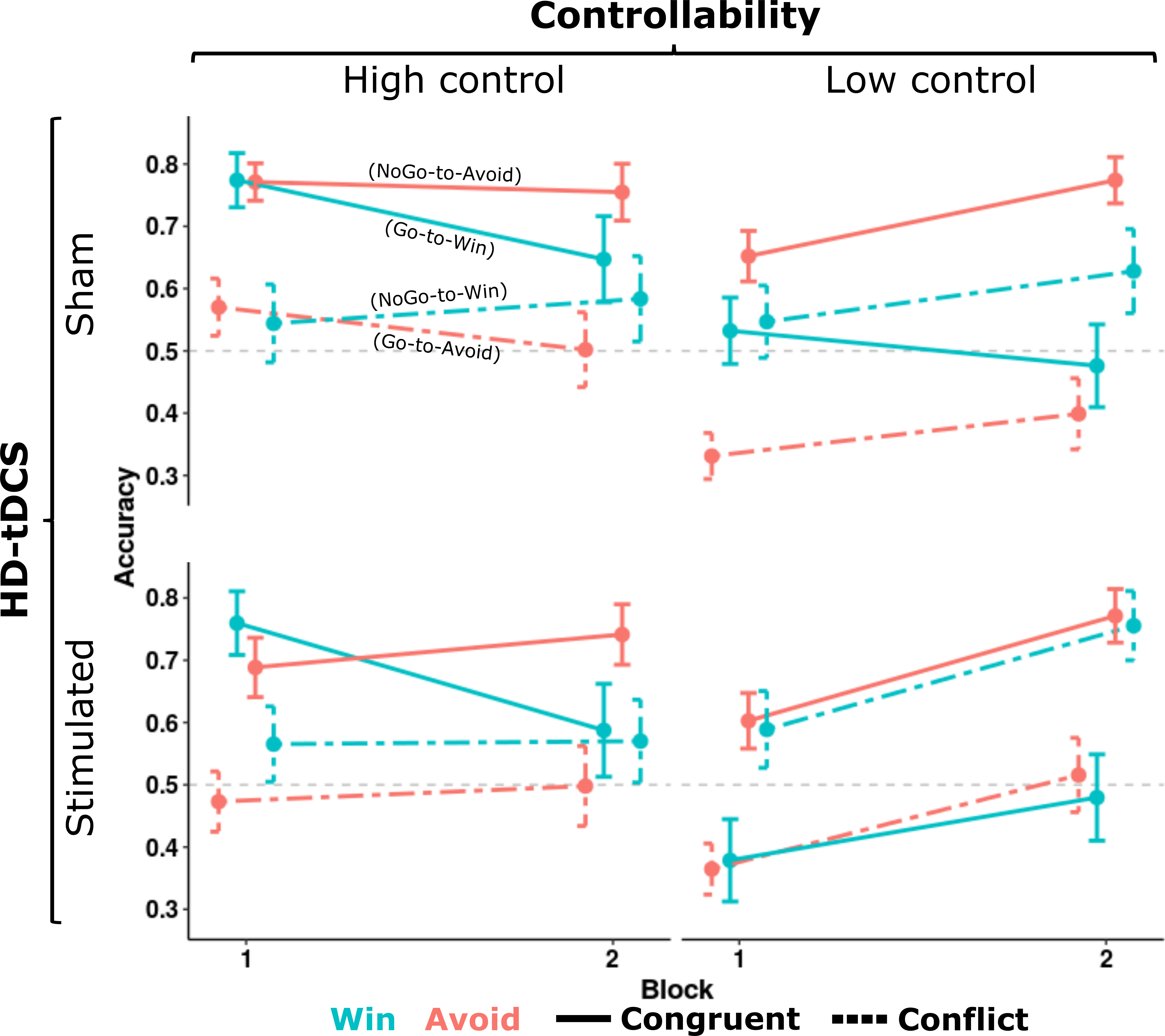

Supplement: Extended Data Figure 4-1 — Response accuracy (means and SEs) across the two blocks and four experimental groups, plotted separately for the four card types. Download Figure 4-1, TIF file. [file enu-eN-NWR-0041-21-s03.tif]

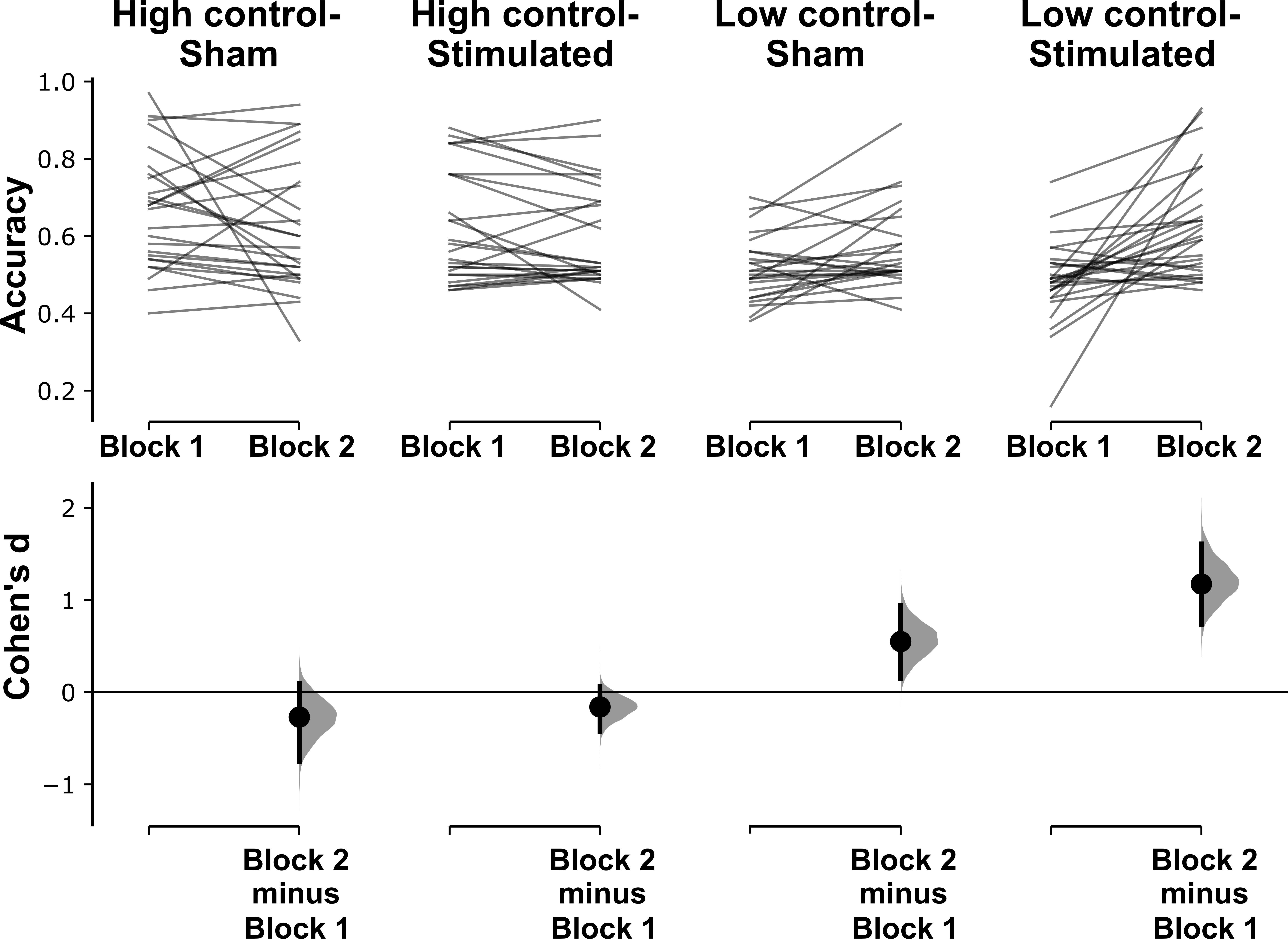

Supplement: Extended Data Figure 4-2 — Raw data (upper panel) showing changes in response accuracy from block 1 to block 2 for each participant, and Cumming estimation plots (lower panel) representing effect size estimates (Cohen’s d) for the change in response accuracy from block 1 to block 2, plotted separately for the four groups. Mean differences are presented as dots, along with the corresponding bootstrap sampling distributions (5000 samples) and the bias-corrected and accelerated 95% CIs. Download Figure 4-2, TIF file. [file enu-eN-NWR-0041-21-s04.tif]

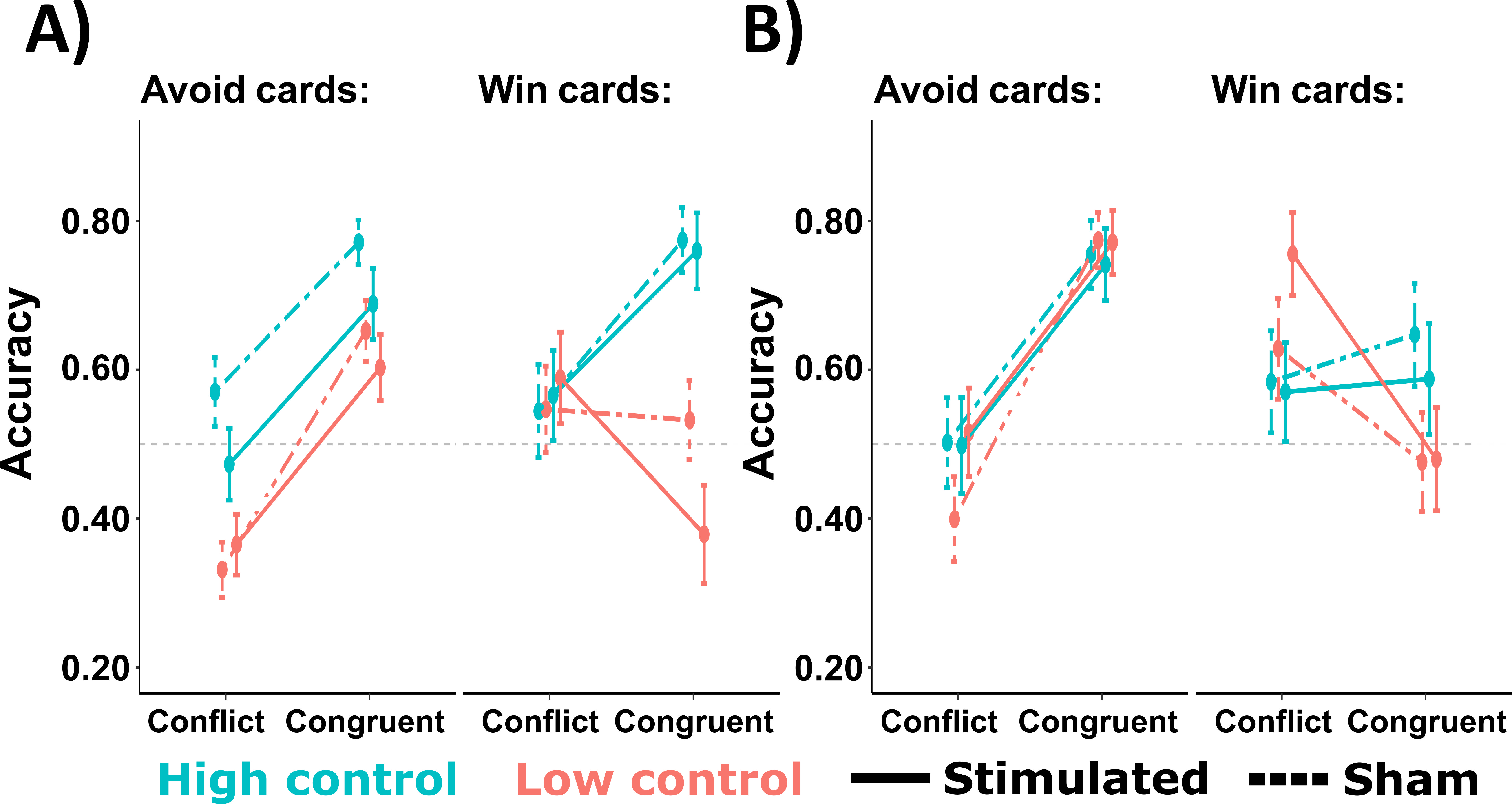

Supplement: Extended Data Figure 4-3 — Response accuracy for Pavlovian-congruent and conflict cards, plotted separately for Win and Avoid cards for block 1 (A) and block 2 (B). Please note that the concept of Pavlovian congruency cannot be interpreted in block 1 for the LowControl groups, as it just reflects the arbitrary labeling of the two Win cards as Go-to-Win and NoGo-to-Win, without underlying response-feedback contingency that could drive cue-response learning. Download Figure 4-3, TIF file. [file enu-eN-NWR-0041-21-s05.tif]

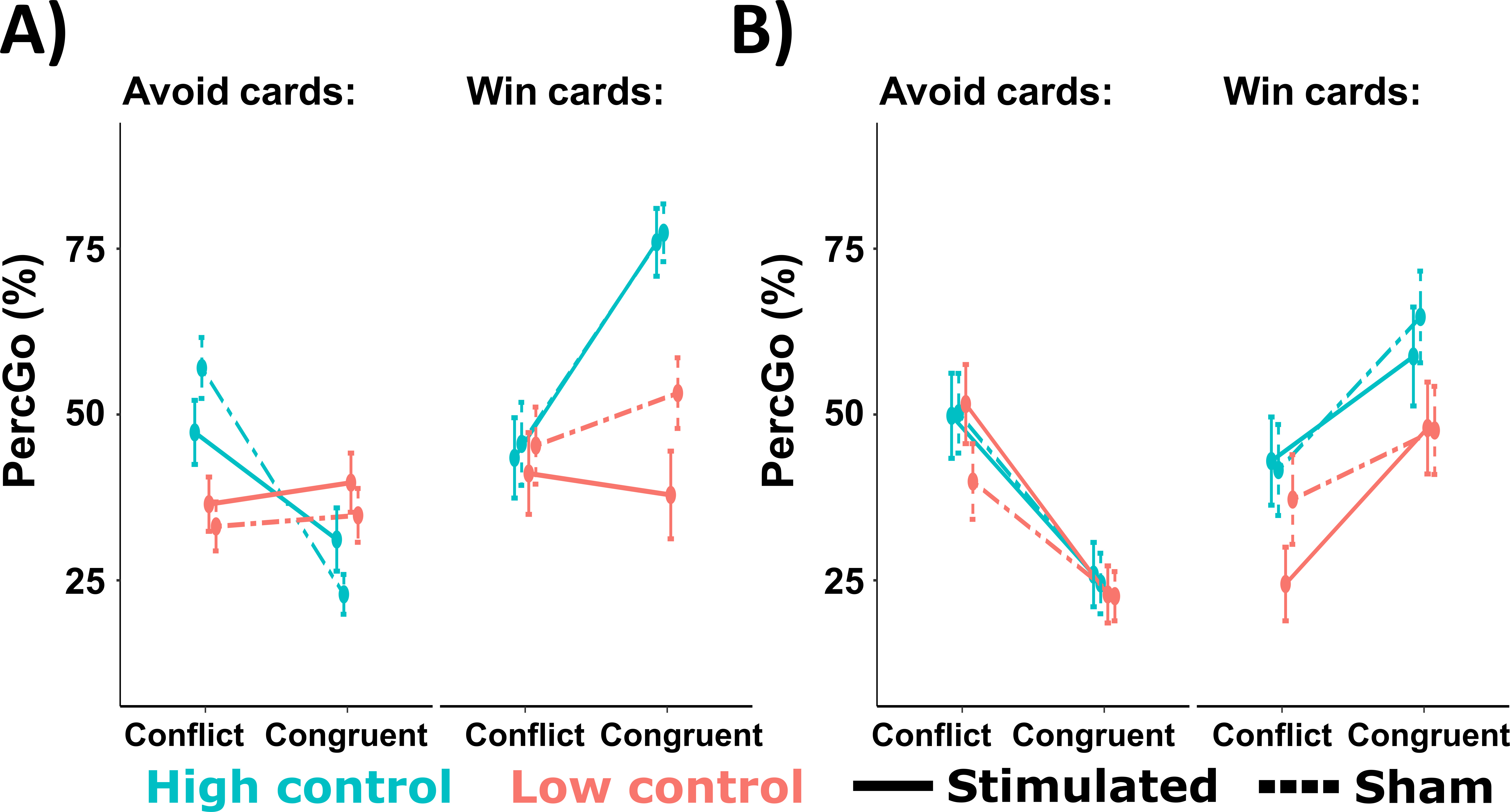

Supplement: Extended Data Figure 4-4 — Percentage of Go responses (PercGo) for Pavlovian-congruent and conflict cards, plotted separately for Win and Avoid cards for block 1 (A) and block 2 (B). Please note that the concept of Pavlovian congruency cannot be interpreted in block 1 for the LowControl groups, as it just reflects the arbitrary labeling of the two Win cards as Go-to-Win and NoGo-to-Win, without underlying response-feedback contingency that could drive cue-response learning. Download Figure 4-4, TIF file. [file enu-eN-NWR-0041-21-s06.tif]
